# YACHT: an ANI-based statistical test to detect microbial presence/absence in a metagenomic sample

**DOI:** 10.1093/bioinformatics/btae047

**Published:** 2024-01-24

**Authors:** David Koslicki, Stephen White, Chunyu Ma, Alexei Novikov

**Affiliations:** Department of Computer Science and Engineering, Pennsylvania State University, State College, PA 16802, United States; Department of Biology, Pennsylvania State University, State College, PA 16802, United States; Huck Institutes of the Life Sciences, Pennsylvania State University, State College, PA 16802, USA; One Health Microbiome Center, Pennsylvania State University, State College, PA 16802, United States; Department of Mathematics, Pennsylvania State University, State College, PA 16802, United States; Huck Institutes of the Life Sciences, Pennsylvania State University, State College, PA 16802, USA; Department of Mathematics, Pennsylvania State University, State College, PA 16802, United States

## Abstract

**Motivation:**

In metagenomics, the study of environmentally associated microbial communities from their sampled DNA, one of the most fundamental computational tasks is that of determining which genomes from a reference database are present or absent in a given sample metagenome. Existing tools generally return point estimates, with no associated confidence or uncertainty associated with it. This has led to practitioners experiencing difficulty when interpreting the results from these tools, particularly for low-abundance organisms as these often reside in the “noisy tail” of incorrect predictions. Furthermore, few tools account for the fact that reference databases are often incomplete and rarely, if ever, contain exact replicas of genomes present in an environmentally derived metagenome.

**Results:**

We present solutions for these issues by introducing the algorithm YACHT: **Y**es/No **A**nswers to **C**ommunity membership via **H**ypothesis **T**esting. This approach introduces a statistical framework that accounts for sequence divergence between the reference and sample genomes, in terms of ANI, as well as incomplete sequencing depth, thus providing a hypothesis test for determining the presence or absence of a reference genome in a sample. After introducing our approach, we quantify its statistical power and how this changes with varying parameters. Subsequently, we perform extensive experiments using both simulated and real data to confirm the accuracy and scalability of this approach.

**Availability and implementation:**

The source code implementing this approach is available via Conda and at https://github.com/KoslickiLab/YACHT. We also provide the code for reproducing experiments at https://github.com/KoslickiLab/YACHT-reproducibles.

## 1 Introduction

### 1.1 Background and context

In metagenomics, that is, the study of microbial communities via their environmentally sampled DNA, there has been increasing interest in the so-called “rare microbiome” (Sogin *et al.* 2006, Reveillaud *et al.* 2014, Ainsworth *et al.* 2015, Noecker *et al.* 2017, Rocca *et al.*, 2020, Cao *et al.* 2021). These are microbial organisms or taxa with low relative abundance in a given sample where many ecologically important microbes are found (Jousset *et al.* 2017). However, computational approaches to inferring the presence and relative abundance of microbes or taxa in given metagenomic sample, so-called “profiling techniques” often suffer from the inability to distinguish between statistical noise and microbes at low relative abundance (Mande *et al.* 2012, Shakya *et al.* 2013, Sczyrba *et al.* 2017, Meyer *et al.* 2022). As a result, practitioners often resort to filtering their profiles based on some abundance threshold: ignoring tool predictions of taxa with relative abundance below some threshold (Simon *et al.* 2019), or else removing the lowest abundance predictions until some fixed amount of abundance has been removed (Meyer *et al.* 2022). Such approaches are ad hoc with little guidance in how to choose thresholds (Cao *et al.* 2021) and have demonstrated negative effects on community analysis and interpretation (Schloss 2020, Jia *et al.* 2022) as well as on profiling tool performance (Meyer *et al.* 2022). Sophisticated filtering approaches exist but are usually limited to specific numeric quantities of interest (such as preserving mutual information (Mokhtari and Ridenhour 2022) or maximizing covariance (Smirnova *et al.* 2019)) or else ignore biologically relevant considerations such as molecular evolution, sequencing error, and low sequencing sampling rates (with some exceptions, including Piro *et al.* (2016)). Current microbiome practitioners thus have few biologically interpretable tools at their disposal to confidently claim a particular microbe is present at low abundance in their sample, or else reject it as noise. This has negative impacts on the study of the rare microbiome and has even resulted in high-profile cases of misinterpreting the presence or absence of microbes in a sample (e.g., Plague in the New York subway system (Ackelsberg *et al.* 2015, Afshinnekoo *et al.* 2015, Gonzalez *et al.* 2016)).

We address this situation by developing a theoretically sound statistical test for the presence or absence of a microbe in a metagenomic sample while accounting for sequencing depth constraints and evolutionary divergence between reference and sample genomes. We describe and mathematically characterize the method and demonstrate its effectiveness with experiments based on both simulated and real-world data.

### 1.2 Summary of approach

We now provide a high-level idea of the approach and its biological interpretation. Two recent innovations facilitated this approach: (i) a statistical framework in which to study sequence evolution via alignment-free, *k*-mer-based approaches (Blanca *et al.* 2022) and (ii) a novel data reduction technique (Hera *et al.* 2023) and associated metagenomic profiler (Irber *et al.* 2022). The former allows us to relate *k*-mer-based statistics (such as the Jaccard or containment index) to biologically relevant quantities such as average nucleotide identity (ANI) (Konstantinidis and Tiedje 2005a, b) while also providing associated confidence intervals and hypothesis tests. The latter innovation provides us a means to quickly derive the prerequisite *k*-mer-based quantities while also providing a theoretical framework in which hypothesis tests can be developed.

Our approach requires the following information from a user: first, a specified false negative rate 1−α which controls the sensitivity of predicting microbial presence or absence. Next the user indicates at what value *A* of ANI they consider two organisms to be biologically identical, which allows us to account for evolutionary sequence divergence between reference and sample genomes. Lastly, the user indicates what fraction *c* of the genetic material exclusive to a given microbe’s genome needs to be present in the sample (i.e. minimum coverage (Sims *et al.* 2014)) for the microbe itself to be called “in the sample.”

The method uses a sketched reference database of microbial genomes and a sketched metagenomic sequencing sample. These sketches can be generated using sourmash (Brown and Irber 2016, Pierce *et al.* 2019). The method first processes the sketched reference database by removing “duplicate” genomes (i.e. genomes within the ANI threshold *A* to another genome in the reference database). Then, it removes organisms in the reference database that have no *k*-mer overlap with the given sample based on a given *k* (e.g., k=31,51, etc.). Among these candidate microbes, the method then identifies which *k*-mers are exclusive to each genome. A hypothesis test is then performed for each candidate genome that answers the question:

Does the sample contain enough of the *k*-mers exclusive to this reference genome to support the claim that an organism, with ANI at least *A* to this reference genome, is contained in the sample with coverage at least *c*?

If the answer is in the affirmative, then this reference organism is said to be *present* in the sample. Importantly, this process is conducted in an alignment-free fashion, facilitating large-scale analyses and ever-expanding reference databases.

Here we use the term “hypothesis test” to mean a test in which only one “null” hypothesis is considered and statistical evidence is weighed to decide whether or not to reject this hypothesis. This type of statistical test is sometimes interchangeably referred to as a “significance test” Fisher (1955); these two outcomes motivate the “yes” and the “no,” respectively, in the name of our approach.

### 1.3 Contribution

To our understanding, this is the first approach that disentangles the difference between low-abundance sample organisms and those that are diverged from a given reference database. Low-abundance organisms present in a sample often have only a fraction of their genome covered by sample reads. Our approach’s ANI threshold *A* and coverage parameter *c* account for this. Hence, as long as *c* percentage of a genome’s unique *k*-mers are covered by at least one read, confidence can be had as to this genome’s presence or absence in the sample. Our experiments (see Fig. 4) demonstrate that *c* can reliably be set lower than 10%, which translates to relative abundances as low as 0.0035%, though false positives start to increase for c<0.05.

A drawback of this method is that genomic evolution and sequencing errors cannot be disentangled. Indeed, from a *k*-mer perspective and the mutation model we assume, there is no difference between “the similarity of one genome and a 1−A-mutated version of it,” and “the similarity of one genome and a 1x-coverage sampled version of it that has a sequencing error rate of 1−A.” Fortunately though, *k*-mers that have been created due to sequencing error do not factor into the determination of presence or absence of organisms in a sample for sufficiently large *k*, since only unique *k*-mers matching to the reference are considered in the hypothesis test.

This method also addresses incomplete reference databases, known to hinder metagenomic profiling and analysis (Kunin *et al.* 2008, Schlaberg *et al.* 2017, Loeffler *et al.* 2020, Marcelino *et al.* 2020), by only requiring a mutated version of an organism within the ANI threshold for detection to be possible.

An additional drawback of this approach is that, in contrast to clade-specific marker gene approaches (Segata *et al.* 2012, Sunagawa *et al.* 2013, Milanese *et al.* 2019) or clade-specific *k*-mer based approaches (Lu *et al.* 2017, Wood *et al.* 2019), our method, when used as a taxonomic profiler, does not attempt to classify to higher, internal taxonomic ranks. This is due to the fact that average nucleotide (or amino acid) identity does not harmonize with taxonomy, though there have been efforts to re-define taxonomy to address this discrepancy (Chaumeil *et al.* 2020, Parks *et al.* 2022). Thus, we focus on ANI similarity rather than taxonomic similarity.

Our framework extends beyond detecting organisms in metagenomic samples, needing only a reference database and a sample with mutated or partially sampled reads. It is thus straightforward to extend this approach to other applications such as functional annotation (where the reference consists of genes or protein families, and the sample is inferred protein-coding sequences), metatranscriptomics (where the reference consists of transcripts and the sample is a shotgun RNA-seq sample), etc.

## 2 Algorithm

In this section, we introduce the YACHT algorithm for detecting organisms in a metagenomic sample and the associated mathematical model.

### 2.1 Preliminaries

We now place the problem of metagenomic presence/absence detection in a mathematical setting. We begin by establishing the following parameters for our model:

-
k∈N , the *k*-mer size,
-
K∈N , the total number of *k*-mers sampled across all organisms,
-
N∈N , the number of known reference genomes,
-
A∈(0,1) , the ANI threshold above which organisms are said to be identical.

The intuition and parameter tuning behind the YACHT algorithm are based on the simple mutation model outlined in (Blanca *et al.* 2022): given a specified ANI *a* and string of nucleotides *S* (the genome), a mutation occurs independently at each site with probability 1−a, in which case that nucleotide is changed uniformly at random into one of the other three.

We assume knowledge of a reference database $G={\left\{G_{i}\right\}}_{i=1}^{N}$ of genomes’ *k*-mers, where each $G_{i}$ is the set of all *k*-mers of the *i*-th reference genome. In practice, computational necessity means we observe only a “sketch” consisting of subsets of each $G_{i}$. By a minor abuse of notation, we will use $G_{i}$ to represent the set of sketched *k*-mers. We use FracMinHash (Hera *et al.* 2023, Irber *et al.* 2022) as the sketching approach since it has the desirable feature that if a *k*-mer appears in two genomes *i* and *j* and is in the sketch $G_{i}$ then it will also be in the sketch $G_{j}$. FracMinHash has a “scale factor” parameter *s* which represents the fraction of *k*-mers that are sampled by the sketch, hence 0<s≤1. A FracMinHash sketch of *k*-mers is a uniformly random selection of s% of those *k*-mers. This is achieved by taking a fixed, uniform hash function *h* with a domain of all *k*-mers and range $\left[0,4^{k}\right]$ and selecting those *k*-mers *x* such that $h(x)\leq s4^{k}$.

### 2.2 Mathematical model for the sample S

Under the aforementioned simple mutation model, so long as the scale factor *s* is relatively small and *k* is relatively large, we have the following consequences, which hold with high enough probability that any deviations can be treated as tolerable noise:

- **Uniqueness:** Each *k*-mer appears at most once in any genome.
- **Non-overlap:** Sketched *k*-mers in the reference do not overlap in the genome from which they were derived. Accordingly, the event of a mutation in each sketched *k*-mer will be independent.
- **One-way Mutation:** If a *k*-mer is not in the sketch, the mutation process does not mutate it into a *k*-mer that is in the sketch. Therefore mutation causes *k*-mers to drop out of the sketch, but not to enter it.

Note: picking *s* too large and/or *k* too small will violate the above assumptions and will reduce the power of this approach, possibly introducing a higher false negative rate as well. Clearly, the assumption of only point mutations is biologically unrealistic, but we later see in numerous experiments that this assumption is not very detrimental to the approach.

Given a genome sketch *G* we can model the sketch $G^{a}$ of a *mutated copy* of *G* with ANI *a* to the original genome as follows: for each *k*-mer g∈G, mutate each site with probability 1−a, which means each *k*-mer will be mutated with probability $1-a^{k}$. By non-overlap, these mutations will be independent. If the *k*-mer *g* is mutated, by one-way mutation, the mutated version will not be in the sketch; moreover, by uniqueness this was the only instance of *k*-mer *g* in the genome represented by *G* and so *g* does not appear in the resulting mutated sketch. Thus the sketch $G^{a}$ of the mutated genome can be characterized by the process: for each g∈G, mutate it with probability $1-a^{k}$. Then $G^{a}$ consists of the set of all *k*-mers which survive this process, i.e. which are *not* mutated.

Under this definition, the sketch of a random metagenomic sample can be modeled as the union of many such sketches of randomly mutated copies of known genomes. More precisely:

Definition 1 (**Randomly Mutated Sketch**). Given *k*-mer size *k*, ANI a∈[0,1], and finite set of *k*-mers *G*, let ${\left\{\chi_{g}\right\}}_{g\in G}$ be a set of independent Bernoulli random variables taking values in {0,1} with $P\left(\chi_{g}=1\right)=a^{k}$. We define the sketch of a randomly *a*-mutated copy of *G* as the random set $G^{a}=\{g\in G|\chi_{g}=1\}$.

In words, $G^{a}$ is the set of unmutated *k*-mers in *G* after undergoing the simple mutation process with ANI *a*. Accordingly, if *G* is a sketch of *k*-mers from a genome, then $G^{a}$ represents the sketch of *k*-mers from a mutated version of that genome with expected ANI *a* to the unmutated version. Similarly, we posit the following mathematical model for the sample S:

Definition 2 (**Randomly Mutated Sample Sketch**). Given *k-*mer size *k* and an *N-*element set of ANI’s a, let G be a collection of *N* finite sets of *k-*mers $G_{i}$*.* Then the sketch of a randomly a-mutated sample $S^{a}$ is defined as the union of $a_{i}$*-*mutated copies of $G_{i}$*:*

$$S^{a}=\overset{N}{\underset{i=1}{\cup }}G_{i}^{a_{i}}.$$

In short, given a set of ANI’s a, each genome’s set of *k*-mers $G_{i}\in G$ is mutated according to the ANI $a_{i}$ into a new sketch $G_{i}^{a_{i}}.$ This definition accounts for the case that only a subset of genomes in G are in the sample by setting $a_{i}=0$ for any genomes which are not in the sample, as these will never have any *k*-mers appearing in $S^{a}$ with this model.

The problem of organism membership detection in a sample community can thus be modeled as follows: given a pre-selected ANI threshold *A*, reference sketch G, and random sample $S^{a}$, determine for each *i* whether a mutated version of genome *i* is in the sample with ANI at least equal to the given threshold: $a_{i}\geq A$.

### 2.3 The YACHT algorithm: N=1

We first consider the case where our reference G consists of only a single genome sketch *G* containing K k-mers. In this case, under the random sample model in Definition 2, our sample sketch S is equal to the sketch of mutated copy of *G* with an unknown ANI *a*, that is: $S^{a}=G^{a}$.

Since all *k*-mers in each genome are assumed to have the same mutation probability, only the set size $\left|S^{a}\right|$ is useful to us, not the identities of the *k*-mers in $S^{a}$. Under the model of Definition 2 with N=1, $|S^{a}|=|G^{a}|=\sum_{g\in G}\chi_{g}$. Accordingly, $\left|G^{a}\right|$ is distributed as a binomial random variable with *K* trials and success probability $a^{k}$. We denote this distribution as $\text{Binom}(a^{k},K)$.

Our goal, as before, is to determine whether a≥A, which is equivalent to testing whether $a^{k}\geq A^{k}$. We can thus reduce the problem to one of testing whether $a^{k}<A^{k}$ based on a single sample $\left|S^{a}|∼\text{Binom}(a^{k},K\right)$. This is a classical problem of hypothesis testing, a field famously studied by Neyman and Pearson (1933). Neyman and Pearson identified that to appropriately perform hypothesis testing, one must separate errors into two types: *type I errors* in which the test incorrectly rejects a null hypothesis (*H*0) that is actually true; and *type II errors*, in which the test fails to reject the null hypothesis when it is false. Accordingly, the established approach when constructing such tests is to choose a *significance level* (one minus the probability of error) for one of the two types, and then to endeavor to minimize the probability of the other.

We pursue such an approach here, choosing to specify the probability of false negative (the failure to report the presence of an organism in the sample). To do this, we define the *hypothesis boundary* as follows:

Definition 3 (**Hypothesis Boundary**). The hypothesis boundary for n k-mers with *k*-mer size *k*, ANI threshold *A*, and significance level α is denoted q(n,k,A,α) and is defined as the largest integer *q* satisfying P(X≥q)≥α for a random variable $X∼\text{Binom}(A^{k},n)$.

In biological terms, given a set of *k*-mers *G*, q(|G|,k,A,α) is the greatest number such that the sketch of a randomly mutated copy of *G* with ANI exactly *A* will contain at least q(|G|,k,A,α) unmutated *k*-mers with probability at least α. Therefore, by definition, given α, the test that rejects the null hypothesis whenever |S|<q(|G|,k,A,α) and accepts it otherwise, is guaranteed to have a type I error probability of at most 1−α; see Algorithm 1. Biologically, this corresponds to a maximum false negative probability of 1−α, where a “false negative” refers to the test rejecting the presence of an organism that is actually in the sample (recall the null hypothesis is “the organism is present in the sample”). Moreover, this holds true with no distributional assumptions on the ANI *a* (more powerful tests exist if one is willing to make distributional assumptions on the ANI *a*, but these will be highly dependent on the underlying biological system).

Biologically speaking, we claim that our reference genome is present whenever the sample shares at least q(|G|,k,A,α) k-mers with the reference genome; when fewer than q(|G|,k,A,α) shared *k*-mers are present in the sample, we conclude that too many *k*-mers have mutated for the sample genome to be related to the reference at the *A* ANI level.

Algorithm 1 YACHT_N1, N=1

**Input:** Significance level α, ANI threshold *A*, *k*-mer size *k*, reference genome *G*, sample S.

**Output:** boolean indicating whether or not the reference genome is in the sample.

1: Set q=q(|G|,k,A,α), the hypothesis boundary (3) for |G| k-mers with *k*-mer size *k*, ANI threshold *A*, and significance level α.

2: Return bool(|S|≥q)

### 2.4 Accounting for coverage

The current model assumes *k*-mers are absent from S only due to mutations, effectively assuming that coverage (the proportion of *k*-mers from the genome recorded in the sample) is 1, which is often not the case due to varying sequencing depth.

Accounting for coverage requires trade-offs: for instance, to guarantee a fixed rate of false negatives in the presence of arbitrarily small coverage would require accepting the presence of organisms sharing even a single *k*-mer with the sample. To avoid introducing distributional assumptions on the coverage parameter, we introduce a user-specified minimum coverage parameter C∈(0,1] and calibrate the hypothesis boundary in Algorithm 1 to guarantee significance α for any organism in the sample that has at least a *C*-fraction of its exclusive *k*-mers present in the sample. The resulting algorithm, detailed in Algorithm 2, is the same as Algorithm 1 with |G| replaced by ⌊C|G|⌋ in the computation of the hypothesis boundary *q*, where ⌊⋅⌋ is the integer floor function.

Algorithm 2 YACHT_N1, N=1

**Input:** Significance level *α*, ANI threshold *A*, *k*-mer size *k*, minimum coverage *C*, reference genome *G*, sample S

**Output:** boolean indicating whether or not the reference is in the sample

1: Set q=q(⌊C|G|⌋,k,A,α), the hypothesis boundary (3) for |G| k-mers with *k*-mer size *k*, ANI threshold *A*, and significance level α.

2: Return bool(|S|≥q)

### 2.5 YACHT: General case, N≥1

In the general case, we cannot employ the same algorithm as in the N=1 case due to the issue of shared *k*-mers between genomes. Unmodified application of Algorithm 2 would allow for the appearance of *k*-mers in the sample to support the presence of multiple organisms in the reference, leading to potential false positives.

However, due to the properties of metagenomic data, we are able to employ Algorithm 1 with only minor modifications. In practice, the sketches even of closely related genomes will still contain a significant proportion of *k*-mers exclusive to each genome (that is, *k*-mers which are not contained in any other genome sketch $G_{i}$), as long as *k* is sufficiently large. Thus we can restrict application of Algorithm 2 only to those *k*-mers which are not shared by multiple genomes in the reference. Mathematically speaking, we employ the following definition:

Definition 4 (**Exclusive *k*-mers**). Given a collection of genomes $G={\left\{G_{i}\right\}}_{i=1}^{N}$, the set ${\tilde{G}}_{i}$ of exclusive *k*-mers of genome *i* is defined as the set of *k*-mers in $G_{i}$ that are not in $G_{j}$ for any j≠i:

$${\tilde{G}}_{i}=G_{i}-\underset{j\in (I-\{i\})}{\cup }G_{j}.$$

We then apply Algorithm 2 to ${\tilde{G}}_{i}$ and the restricted sample $S_{i}={\tilde{G}}_{i}\cap S$. By only considering exclusive *k*-mers, the decision to include each genome has no effect on the inclusion of the others, allowing us to perform the hypothesis test independently and iteratively on each. By construction, this has no effect on the accuracy of tests for individual organisms (aside from the effects of testing with fewer *k*-mers due to only using each organisms’ exclusive *k*-mers).

We pre-filter our reference genomes by removing those that share no *k*-mers with the sample, as they will never be deemed “present,” avoiding interference with non-filtered genomes. The result is the YACHT algorithm detailed in Algorithm 3. We note that algorithm 3 assumes the same minimum coverage for every organism, but there is no reason this parameter cannot be specified per organism.

Algorithm 3 YACHT

**Input:** Significance level α, ANI threshold *A*, *k*-mer size *k*, minimum coverage *C*, collection of reference genomes G, sample S.

**Output:** Vector x with Boolean entries $x_{i}$ indicating whether or not genome $G_{i}$ is in the sample above the ANI threshold *A*.

1: Create x, a N×1 vector of zeros

2: Set $I=\{i\in \{1,\ldots ,N\}\;s.t.\;|G_{i}\cap S|>0\}$

3: For i∈I, set ${\tilde{G}}_{i}=G_{i}-\underset{j\in (I-\{i\})}{\cup }G_{j}$

4: **for**i∈I**do**

5:   Set $S_{i}={\tilde{G}}_{i}\cap S$

6:   $x_{i}=\text{YACHTC}\_N1(\alpha ,k,C,{\tilde{G}}_{i},S_{i})$

7: **end for**

8: return x

### 2.6 Statistical power

Since the probability of false negative error is fixed by our test design, the appropriate measure of the effectiveness of a hypothesis test is its *power*: the probability that, given the null hypothesis is false, the test actually rejects the null hypothesis. Although the probability of false negatives is fixed by the parameter α, the power of YACHTC_N1 will vary between organisms in the reference depending on the number of exclusive *k*-mers each has, with organisms with more exclusive *k*-mers enjoying a lower probability of false positives.

Computing YACHT’s power exactly would require a precise distribution for ANI’s, which we have deliberately avoided due to this distribution being highly dependent on the biological situation to which this algorithm is applied. As a distribution-free proxy, we can instead compute the *alternative significance ANI* $a_{i}^{\alpha }$:

Definition 5 (**Alternative Significance ANI**). Given a collection of genomes G, sample S, and significance level α, the alternative significance ANI for organism *i*, denoted $a_{i}^{\alpha }$, is the unique solution to the equation

(1) $$P\left(|S_{i}|\geq q_{i}\left(|{\tilde{G}}_{i}|,k,A,\alpha \right)|a_{i}=a_{i}^{\alpha }\right)=1-\alpha .$$

*In other words*, $a_{i}^{\alpha }$*is the ANI such that if* $a_{i}^{\alpha }$*is the true ANI of organism i, the probability of YACHT returning a false positive is exactly the same as the false negative rate* 1−α.

Equation 1 can easily be solved numerically due to the monotonicity of $P\left(|S_{i}|\geq q\left(|{\tilde{G}}_{i}|,k,A,\alpha \right)|a_{i}=a_{i}^{\alpha }\right)$ as a function of $a_{i}^{\alpha }$. It is also the case that this monotonicity guarantees $a_{i}^{\alpha }\leq A$, and it implies that any genome with an ANI less than $a_{i}^{\alpha }$ will be even less likely to appear as a false positive. The closer $a_{i}^{\alpha }$ is to the ANI threshold *A*, the narrower the range in which YACHT is likely to make a false positive judgment and so the greater the statistical power.

### 2.7 Practical considerations

Algorithm 3 can be run for any set of reference genomes G. Good practical performance will only be achieved if each genome has an appreciable number of exclusive *k*-mers, which requires that no two reference genomes share essentially all of their *k*-mers. Accordingly, we employ the following pre-processing algorithm to ensure that no two organisms are more closely related than the species ANI threshold *A*. By a greedy algorithm, we find a *A -distinct subcollection* $G^{A}\subseteq G$ defined as follows:

Definition 6 (***A*-distinct subcollection**). Given *k*-mer size *k*, ANI threshold *A*, and collection of reference genomes G, an *A*-distinct subcollection of G is a collection $G^{A}\subseteq G$ such that for every $G_{i}^{A},G_{j}^{A}\in G^{A}$ with i≠j:

(2) $$\frac{G_{i}^{A}\cap G_{j}^{A}}{\min\{|G_{i}^{A}|,|G_{j}^{A}|\}}\leq A^{k}.$$

In biological terms, whenever two reference organisms are related above the ANI threshold, we choose one to represent that “class” of related organisms. We discard the smaller of any two related genomes, as the larger will typically enjoy greater statistical power with YACHT. The algorithm is detailed in Algorithm 4.

Algorithm 4 YACHT preprocessing

**Input:** *k*-mer size *k*, ANI threshold *A*, collection of reference genomes G.

**Output:** *A*-distinct subcollection $G^{A}\subseteq G$

1: Sort G according to ascending size

2: Set J={1,2,…,N}

3: **for**i=1:N**do**

4:  **for**j∈J−{i}**do**

5:   **if**$\frac{\left|G_{i}\cap G_{j}\right|}{\left|G_{i}\right|}>A^{k}$**then**

6:    J=J−{i}

7:    **break**

8:   **end if**

9:  **end for**

10: **end for**

11: Set $G^{A}=\{G_{j}\in G|j\in J\}$

12: return $G^{A}$

The effects of excluding such a preprocessing step are reflected in Supplementary Fig. S8, which for synthetic data shows a dramatic increase in false positive rate when organisms in the reference are more closely related than the ANI threshold.

## 3 Theory

In this section, we provide theoretical bounds on the hypothesis boundary q(n,k,A,α) as well as the alternative significance ANI $a^{\alpha }$. For simplicity of notation we restrict consideration to the single-genome case, but our results apply equally to the N>1 case under the substitution $G\to {\tilde{G}}_{i}$ and $S\to S_{i}$. To further simplify notation, in this section we will use the following shorthand: n=|G|, q=q(n,k,A,α), and $\mu =nA^{k}$. All proofs are in the Supplementary material.

We begin with the following theorem, which bounds the distance between *q* and the mean number of unmutated *k*-mers μ (see the proof in Supplementary Section S1):

Theorem 1. Fix α∈(0,1). Let *X* be a binomial random variable with probability of success *p*, *n* trials, and mean $\mu =nA^{k}$. Let *q* be the largest integer such that P(X≥q)≥α. Then there exists a positive constant $C_{\alpha }$ depending only on α such that:

$$\mu -C_{\alpha }\sqrt{\mu }\leq q\leq \mu +C_{\alpha }\sqrt{\mu }.$$

As a corollary, we can immediately infer that as *n* becomes large, *q* tends to be close to the mean μ:

**Corollary 1.**μ/q→1 as n→∞.

We now prove a bound relating the alternative significance ANI $a^{\alpha }$ to the ANI threshold *A*:

Theorem 2. Fix α∈(0.5,1) and let *q* be the largest integer such that P(X≥q)≥α for $X∼\text{Binom}(A^{k},n)$. Let $a^{\alpha }$ be the number in (0,A) such that for $Y∼\text{Binom}({\left(a^{\alpha }\right)}^{k},n)$, P(Y≥q)=1−α. Then there exists a positive constant $C_{\alpha }$ depending only on α such that:

(3) $$\gamma_{n,k,A}A\leq a^{\alpha }\leq A$$

where

$$\gamma_{n,k,A}={\left(1-\frac{C_{\alpha }}{\min\left\{nA^{k},\sqrt{nA^{k}}\right\}}\right)}^{1/k}.$$

In particular, as n→∞ with *k* fixed, $a^{\alpha }\to A$.

In words, this means that the statistical power of the test approaches 1 as the number of exclusive *k*-mers increases.

Theorem 2 also suggests the somewhat surprising result that increasing *k* does not monotonically improve accuracy (in the sense of bringing $a^{\alpha }$ closer to *A*). The $\gamma_{n,k,A}$ term in line (3) has a unique global and local maximum for k≥1, suggesting there is an optimal *k* that maximizes the power of YACHT for a specific number of unique *k*-mers *n*. This phenomenon corresponds to the fact that for large *k*, nearly all of a genome’s *k*-mers will be mutated at the ANI threshold *A*, so the hypothesis boundary *q* must be set very close to zero. When this happens, only small deviations are necessary for more highly mutated genomes to cross the inclusion threshold. This behavior is reflected in the Supplementary Fig. S3.

## 4 Experiments

In this section, we conducted a series of experiments utilizing both simulated and real-world data to fully evaluate YACHT. We performed four different experiments: 1) synthetic data-based experiments; 2) spike-in experiments; 3) Critical Assessment of Metagenome Interpretation (CAMI) II-based experiments; and 4) real-world data-based experiments. To illustrate the advantage of YACHT in determining the presence or absence of an organism in a sample, we also compared it with the sub-routines prefetch and gather of sourmash that have the similar functions in the Supplementary Section S4.

### 4.1 Synthetic data-based experiments

We used synthetic data to elucidate the impact of model parameters on performance, as well as validations of the theory; see Supplementary Section S2.

**Figure 1.**
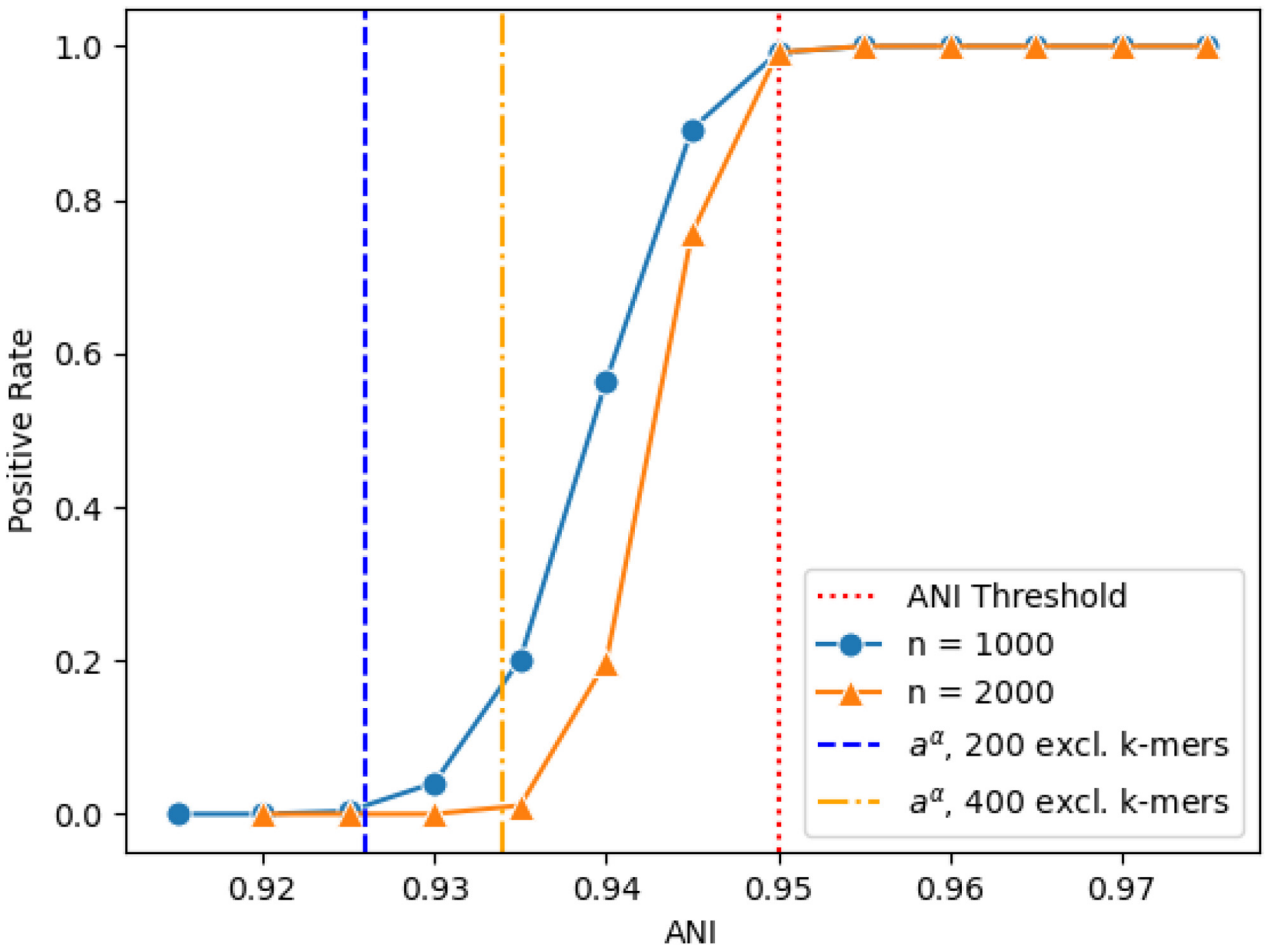
The impact of ANI with different number of kmers on the YACHT result based on synthetic data. The vertical/*y*-axis represents positive rate, defined as the proportion of 100 iterations per organism which YACHT identifies to be “present” in the synthetic sample employing 0.95 as ANI threshold. The horizontal/*x*-axis gives the true ANI rate of each organism in the synthetic sample to a genome in the reference. The *n* denotes the total number of k-mers per organism. The left-most and middle vertical dashed lines give the alternative significance ANI using 200 and 400 exclusive *k*-mers, respectively; the right-most vertical dashed line gives the ANI threshold.

A noteworthy result is illustrated in Fig. 1, which shows the influence of number of *k*-mers and true ANI to a genome in the reference on the performance of the YACHT algorithm, under a fixed ANI threshold of 0.95. In this experiment, 200 randomly chosen organisms with ANIs varying from 0.92 to 0.97 comprise the synthetic sample. Over 100 such samples, each organism is identified as present or absent by YACHT and the proportion of successful “present” identifications is calculated as the positive rate. We also compare the difference in YACHT performance under different number of total *k*-mers.

As intended, we can see that when the true ANI is substantially below the ANI threshold, the positive rate is 0, and it quickly increases to 1 once it surpasses the threshold. This indicates that the YACHT algorithm is sensitive to the ANI threshold and correctly uses it to quantify presence or absence. We also observe that the transition from 0 to 1 occurs more quickly for n=2000 than n=1000, reflecting the increase in statistical power that accompanies a greater number of *k*-mers per organism. The blue and orange vertical lines show the alternative significance ANI ($a^{\alpha }$) for 200 and 400 exclusive *k*-mers, respectively, showing that a greater number of *k*-mers corresponds to $a^{\alpha }$ closer to the ANI threshold. This demonstrates the statistical power of this approach as defined in Definition 5, the accuracy of which is quantified by Theorems 1 and 2. That is, Fig. 1 demonstrates the accuracy of the alternative significance.

### 4.2 Spike-in experiments

The spike-in experiments are designed for testing the impact of two important parameters used in YACHT: —min_coverage and—ani_thresh. We utilized 1,044 bacterial genomes randomly selected from NCBI RefSeq (O’Leary *et al.* 2016) to build the reference database, and a randomly selected, publicly available human gut metagenome data from the ENA (Leinonen *et al.* 2010) as the metagenome sample. To test the—ani_thresh parameter, we collect genomes that are not contained in the reference data, but are at a known ANI to genomes that are in the reference. Hence, We used 28,595 genomes from the GTDB (Chaumeil *et al.* 2020, Parks *et al.* 2022) R07-RS207 genomic representatives as known unknowns that are neither in the sample nor in the reference, but are at a known ANI to genomes in the reference database. More details regarding data description and pre-processing can be seen in Supplementary Section S3.

#### 4.2.1 Coverage parameter testing

**Figure 2.**
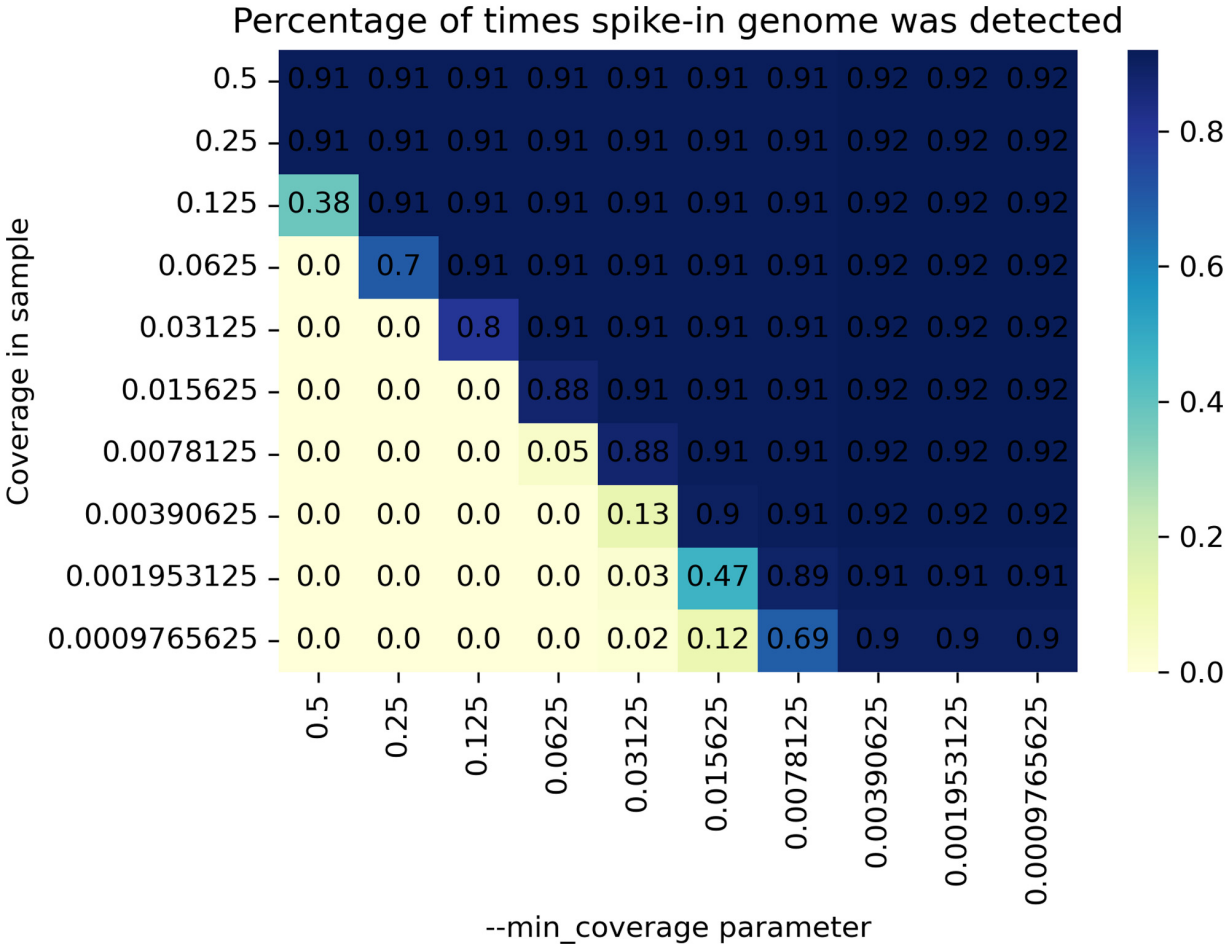
Heatmap of percentage of times the spike-in genome was recovered from the metagenome. The vertical/*y*-axis gives the coverage of the spike-in genome in the metagenome, called *c* in the main text. The horizontal/*x*-axis gives the value of the parameter—min_coverage, called *C* in the main text. Both axes vary by powers of two.

Herein, we aim to test if the parameter—min_coverage allows YACHT to successfully recover a genome from a sample (respectively, correctly report it is absent from the sample) if the coverage is sufficiently high (respectively, if the coverage is too low). Individually for each of the reference genomes that are pre-determined as “absent” from the metagenome sample (see Section S3), we selected error-free, mutation-free reads from it until a total coverage *c* was attained. We then inserted these reads into the metagenome sample and ran YACHT using a—min_coverage value of *C*. The spike-in genome was then labeled “detected” or “not detected” based on the output of Algorithm 3. We then varied the values of *c* and *C* by powers of two and repeated this experiment 100 times, varying the absent reference genomes and averaging results. Figure 2 displays the results as a heatmap. As Fig. 2 demonstrates, our approach successfully recovers a genome from a metagenome if its coverage in the metagenome is at or above the set—min_coverage value with very high probability. The correspondence of the actual coverage of the genome in the metagenome and the—min_coverage parameter is not perfect though, as some percentage of spike-in genomes were detected but had coverage in the sample lower than the set parameter. However, the relationship should be viewed as a lower bound of detection: setting—min_coverage to some value *C* all but guarantees the ability to detect a genome with actual coverage at least *C* in the metagenome.

#### 4.2.2 ANI threshold parameter testing

To assess the impact of the—ani_thresh parameter, we utilize the “known unknown” genomes and spike them in the metagenome sample similar to the previous section. First, we fixed the coverage of each spiked-in genome to c=1 and set the—min_coverage to C=1. We then created 28,595 metagenomes (see Supplementary Section S3), one for each “known unknown” spiked into the metagenome sample, and ran YACHT on each spiked metagenome. For each of these, we recorded if a similar organism was detected or not: that is, if Algorithm 3 detected an genome $G_{i}$ as present in the sample, where $G_{i}$ is the reference database genome with maximal ANI to the spiked-in “known unknown” genome. Of note, the actual relative abundance of the spike-in genomes was at most 0.35% on the basis of reads in the metagenome and from the spike-in genome.

**Figure 3.**
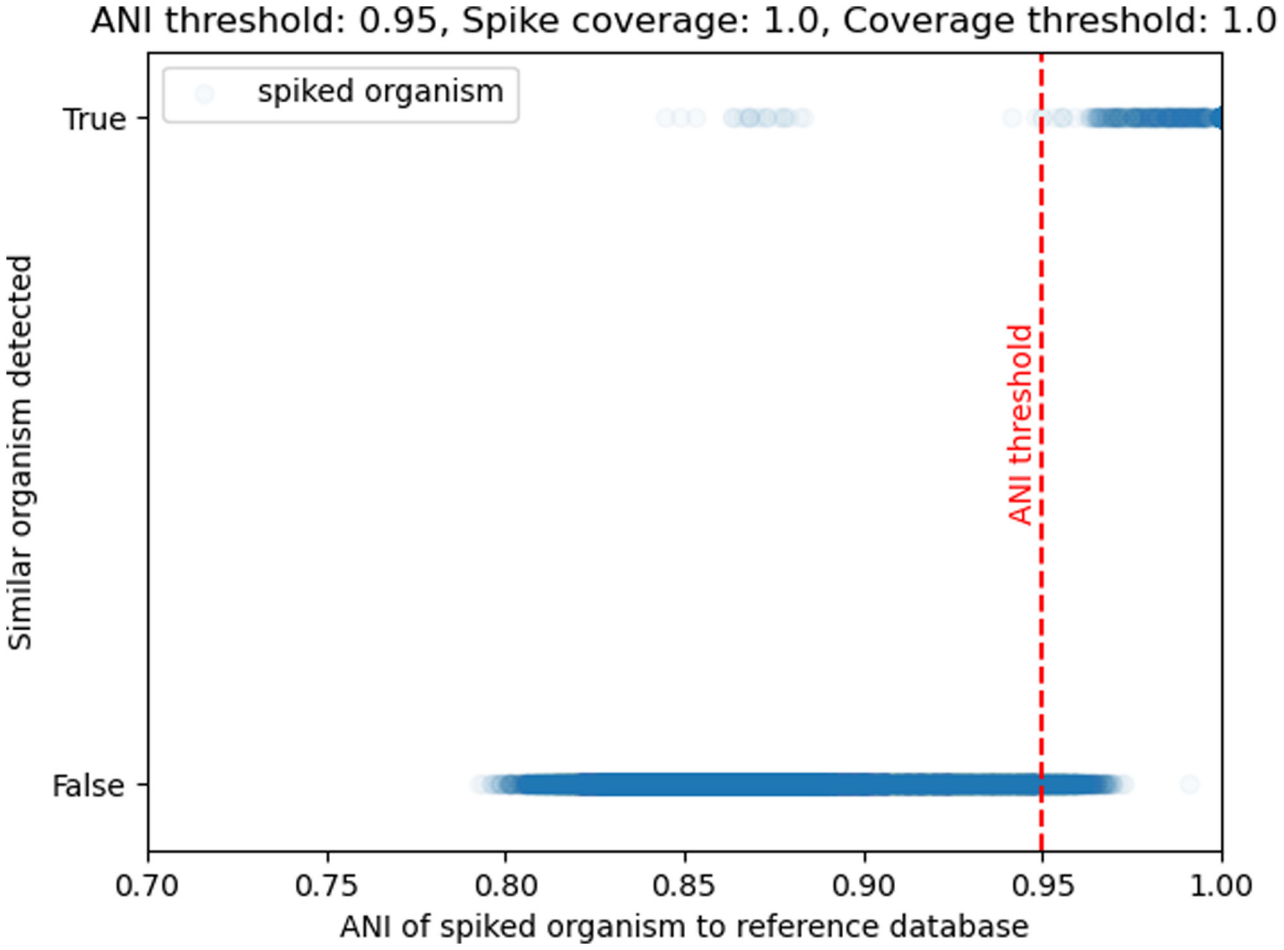
Scatter plot of 28,595 metagenomes, each with a single spiked-in genome absent from the reference database, but with a known maximal ANI to a reference database genome $G_{i}$. This ANI value is recorded on the *x*-axis. The binary *y*-axis records if YACHT reported $G_{i}$ as present (True) or absent (False) and a pale blue dot is recorded accordingly. The red vertical line depicts the setting of the parameter—ani_thresh, here set to an ANI value of A=0.95.

Figure 3 shows the results of this experiment. In more detail, this figure shows that in 97.06% of these experiments, a similar organism was correctly not detected (a true negative) due to the ANI of the spiked organism to the reference being lower than the—ani_thresh value, as seen by the blue dots on the bottom left of the vertical red dashed line. In a total of 99.80% of these experiments, YACHT correctly detected the reference organism similar to the spiked organism (a true positive); the ANI of this pair being above the—ani_thresh parameter, as seen by the blue dots in the top, right of the vertical red dashed line. The false positive cases where the spiked organism had an ANI to the reference genome $G_{i}$ below the ANI threshold, yet YACHT still detected $G_{i}$ can be seen in the top to the left of the vertical red dashed line. We hypothesize that these are due to *k*-mers in the sample that match with those in $G_{i}$ though originating from organisms different from the spike genome. The slightly increased false negative rate of 0.029 over the significance level, which was here set to 0.01 is likely due to violations of our assumptions in Section 2.2 due to real-world mutations not being independent nor one-way.

**Figure 4.**
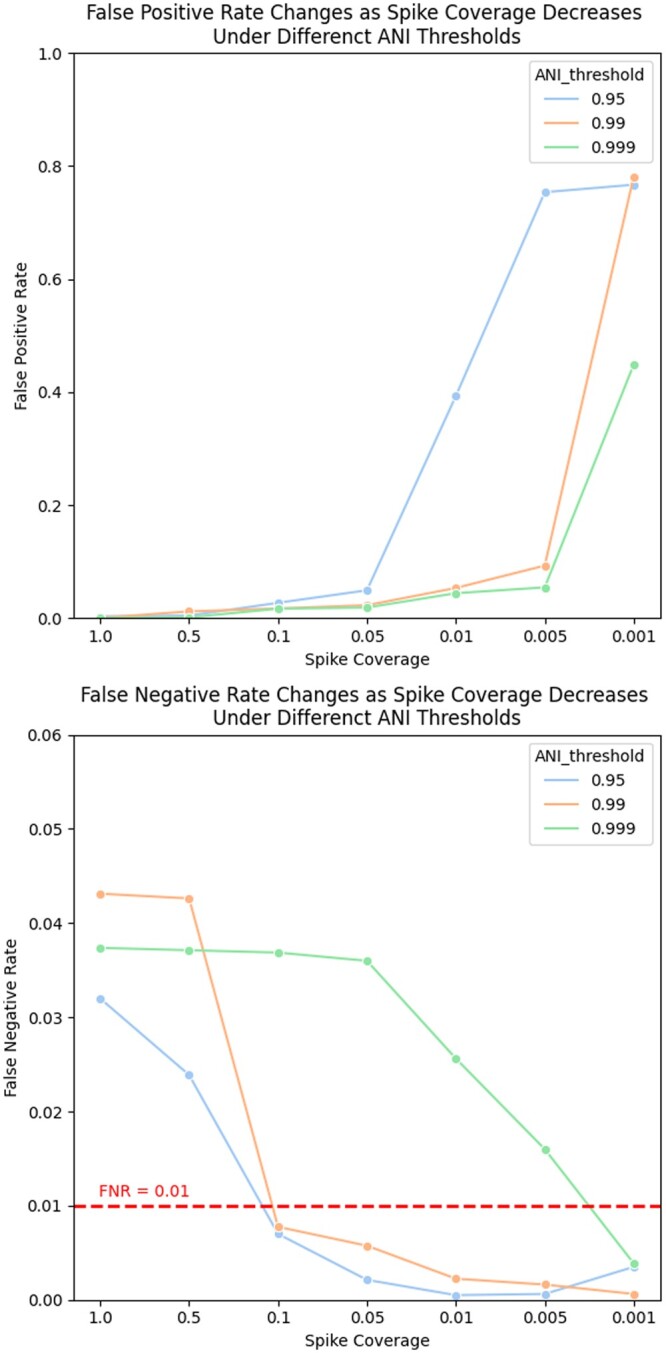
Line plots of false positive rate and false negative rate versus spike coverage under different ANI thresholds. In each plot, the horizontal axis varies the coverage *c* of the spiked in genome in the metagenome (fixing the coverage threshold *C* to *c*), while the vertical axis, respectively, represents false positive rate and false negative rate. We used different color lines to present different ANI threshold settings. In the false positive rate plot, we highlighted the line with FNR = 0.01 with red dash line as α was set to 0.99. Note the different *y*-axes scales in the two figures.

Next, we varied both the—min_coverage parameter *C* and the coverage of the spiked-in genome in the metagenome *c*, while fixing C=c. We used 8K of the spiked metagenomes from the previously described experiment, focusing now on false positives and false negatives. Figure 4 shows the result of these experiments when *c* and *C* both vary from 1.0 to 0.001 while keeping C=c (we also showed the results with C≠c in Supplementary Fig. S11). As these figures demonstrate, decreasing the coverage of the spike-in genome (and decreasing the coverage threshold *c* as well), the false positive rate increases while the false negative rate decreases. This is expected due to a coverage threshold of c=0.001 which corresponds to YACHT needing to see approximately $10^{-6}$ of a genome’s unique *k*-mers to predict it as “present” (due to the scale factor of s=1/1000 and c=0.001). For many organisms, this can be as low as a single *k*-mer or even no *k*-mers if that organism’s genome is small enough. However, we can observe that as the coverage is fixed, increasing the ANI threshold can lower the false positive rate at the cost of increasing the false negative rate. These plots also show that using an ANI threshold > 0.99 and setting coverage *c* between 0.1 and 0.01 appears to balance between both false positive and false negative rates.

### 4.3 CAMI II-based experiments

YACHT can be utilized to solve two tasks in the CAMI II challenge (Meyer *et al.* 2022): taxonomic profiling and pathogen detection, which we report on in this section. We collected the reference genomes from the NCBI RefSeq and Genbank databases, as well as the Bacterial and Viral Bioinformatics Resource Center (BV-BRC) (Olson *et al.* 2023) to build the reference database. For the metagenome samples, we utilized the data provided by the CAMI II challenge: 10 marine, 1 pathogen detection, 21 rhizosphere, and 100 strain madness datasets. More details regarding data description and pre-processing are contained in the Supplementary Section S3.

#### 4.3.1 Presence/absence in taxonomic profiling

**Figure 5.**
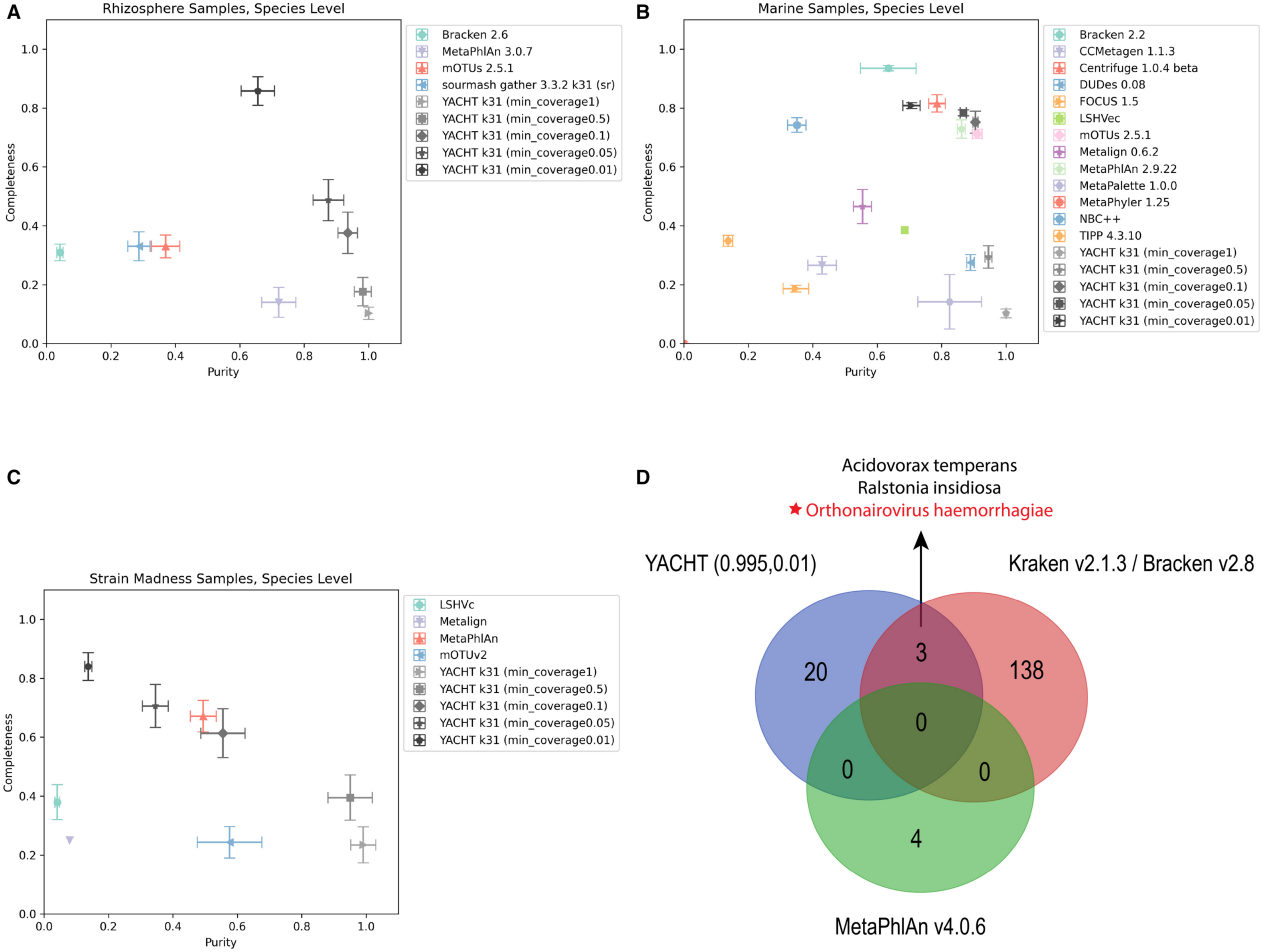
Comparison between YACHT and the SOTA tools reported in CAMI II challenge for the tasks of taxa presence/absence detection and pathogen detection. (A) Using CAMI II Rhizosphere datasets to compare YACHT and the SOTA tools based on completeness and purity metrics; (B) on the Marine datasets; and (C) on the Strain Madness datasets. (D) Comparison between YACHT and the SOTA tools in identifying the causal pathogen: hemorrhagic fever orthonairovirus (CCHFV).

To evaluate YACHT’s performance on the presence/absence portion of taxonomic profiling, we compared it with state-of-the-art (SOTA) methods reported in the CAMI II paper which include Bracken (Lu *et al.* 2017), MetaPhlAn (Segata *et al.* 2012, Beghini *et al.* 2021), mOTU (Milanese *et al.* 2019), LSHvec (Shi and Chen 2021), sourmash_gather, CCMetagen (Marcelino *et al.* 2020), Centrifuge (Kim *et al.* 2016), DUDes (Piro *et al.* 2016), MetaPalette (Koslicki and Falush 2016), Metalign (LaPierre *et al.* 2020), NBC++ (Zhao *et al.* 2020), MetaPhyler (Liu *et al.* 2011), TIPP (Shah *et al.* 2021), and FOCUS (Silva *et al.* 2014). We ran YACHT with five different min_coverage values (i.e. 1, 0.5, 0.1, 0.05, 0.01) on the three types of CAMI II datasets (i.e. marine, rhizosphere, and strain madness), evaluating results with OPAL v1.0.12 (Meyer *et al.* 2019). Figure 5A–C, respectively, show the comparison in completeness (i.e. sensitivity) and purity (i.e. specificity) between YACHT and the SOTA tools on three types of CAMI taxonomic profiling datasets at the species level. As the figures demonstrate, by tuning the parameter—min_coverage between 1 and 0.01, we can balance the performance of YACHT in terms of completeness and purity. YACHT with *min_converage = 0.1* is a consistent top performer in comparison to the SOTA tools on all three types of datasets.

#### 4.3.2 Pathogen detection

Clinical pathogen detection from metagenomics data is an important challenge in computational biology. CAMI II offered a clinical metagenome sample from a blood sample of a patient infected by Crimean–Congo hemorrhagic fever orthonairovirus (CCHFV). The task is to correctly identify the casual pathogen and other potential pathogens. We test if YACHT is able to identify the causal pathogen in this challenge. For comparison, we also include the latest version of two SOTA tools reported in the CAMI II paper: Bracken v2.8 and MetaPhlAn v4.0.6. The results shown in Figure 5D illustrate that YACHT can successfully identify the casual pathogen orthonairovirus haemorrhagiae and other microbes with the settings of *ani_threshold = 0.995* and *min_converage = 0.01*. The method Bracken v2.8 can also correctly identified this causal pathogen and has three total predictions in common with YACHT. Although the CAMI II paper reported that MetaPhlAn can also identify the causal pathogen, we were unable to replicate this via the latest version of MetaPhlAn with default parameters.

### 4.4 Real-world data-based experiment

This section reports on essentially a wet-lab version of the previous spike-in experiments using metagenome data from a previous publication (Costea *et al.* 2017) where they provide a mock bacterial community stool sample composed of 10 known bacterial species and seven individual stool samples spiked with proportions of the mock community. We used the same reference database as we used for the pathogen detection task in the CAMI II-based experiment. More details about data description and pre-processing are shown in Supplementary Section S3.

#### 4.4.1 Target bacteria identification

The goal of this experiment is to test if YACHT can identify those 10 target bacteria reported by the publication from all samples. We found that for the mock community sample, since it was reported to have 10 target bacteria only, the coverage of these species should be comparably large. With *min_converage = 0.5*, YACHT identifies 9 target bacteria while setting *min_converage* to 0.1, YACHT detects all of them. With reduced *min_converage*, YACHT also detects other organisms (such as phages); these non-target microbes are only detected at low coverage and thus are probably contaminants. For those 7 individual samples spiked with mock community, YACHT successfully identifies those 10 target organisms with different *min_converage* settings because the proportions of target organism varies in different samples. We provide all of YACHT’s results for these samples with the metadata of samples and target organism information in the Supplementary materials.

## 5 Conclusion

In this study, we presented YACHT, a statistical test for determining the presence or absence of a genome in a metagenomic sample while also accounting for genome relatedness via ANI, incomplete databases, and genome coverage in the sample. Our synthetic and real-world experiments demonstrate the reliability of YACHT and demonstrate that theoretical predictions extend to real-world data. Additionally, this approach is scalable to large data sets due to its reliance on the sketching technique FracMinHash.

We intend this approach to be useful to biologists in distinguishing between low-abundance microbes in a sample and noise, thus facilitating microbiome analysis with statistical confidence. Additionally, with YACHT, it is no longer necessary to apply ad hoc filtering of low-abundance microbes predicted to be in a sample. Rather users can select a significance level (and hence, desired false negative rate), an ANI threshold where they consider organismal genomes as “essentially the same,” and a coverage value by which a genome is judged as being present in the sample. YACHT will then perform the filtering via its statistical test.

As such, we envision that YACHT can be used as a substitute for sourmash’s prefetch sub-routine. Subsequent application of sourmash’s gather can then be used to associate relative abundances to the genomes deemed present in the sample by YACHT.

## Supplementary Material

btae047_Supplementary_Data

## Data Availability

All the code and example data are available at https://github.com/KoslickiLab/YACHT.
