# Supplementary material for: YACHT: an ANI-based statistical test to detect microbial presence/absence in a metagenomic sample
Source: Bioinformatics. 2024 Jan 24;40(2):btae047. doi: 10.1093/bioinformatics/btae047 (PMC10868342; doi:10.1093/bioinformatics/btae047)
Supplement: btae047_Supplementary_Data [file btae047_supplementary_data.zip › YACHT_bioinformatics_submission_supplement.pdf]

Supplementary material for YACHT: an ANI-based statistical test to detect microbial presence/absence in a metagenomic sample

David Koslicki[1,2,3,4,*,‡], Stephen White[5,*], Chunyu Ma[3,*], Alexei Novikov[5]

[1] Department of Computer Science and Engineering, The Pennsylvania State University
[2] Department of Biology, The Pennsylvania State University
[3] Huck Institutes of the Life Sciences, The Pennsylvania State University
[4] The Microbiome Center, The Pennsylvania State University
[5] Department of Mathematics, The Pennsylvania State University
[*] These authors contributed equally
‡ Corresponding author, dmk333@psu.edu [⋆]

S1 Theoretical Proofs

In this section, we provide theoretical bounds on the hypothesis boundary as well as the alternative significance ANI a^α. For simplicity of notation we restrict consideration to the single-genome case, but our results apply equally to the $N > 1$ case under the substitution $G \to \widetilde{G}_i$ and $\mathcal{S} \to \mathcal{S}_i$. To further simplify notation, in this section we will use the following shorthand: $n = |G|$, $q = q(n, k, A, \alpha)$, and $\mu = nA^k$. In both of our theorems, we make use of Chernoff bounds on sums of Bernoulli random variables. Variants of these are well-known in probability literature; a more precise version, of which the following theorem is a corollary, is proven in [1, Theorem 4.4].

Theorem 1 (Chernoff Inequality). *Let $X_1, \ldots, X_N$ be independent random variables taking values in $\{0,1\}$ with $\mathbb{P}(X_i = 1) = p_i$. Then setting $X = \sum_{n=1}^{N} X_i$ with $\mu = E[X] = \sum_{n=1}^{N} p_i$ and $\delta > 0$, we have:*

1. *If $\delta \in (0,1]$, then $\mathbb{P}(X \geq (1+\delta)\mu) \leq \exp\left(-\mu\delta^2/3\right)$*
2. *If $\delta \in (0,1]$, then $\mathbb{P}(X \leq (1-\delta)\mu) \leq \exp\left(-\mu\delta^2/2\right)$*
3. *If $\delta > 1$, then $\mathbb{P}(X \geq (1+\delta)\mu) \leq \exp(-\mu\delta/3)$*

We begin with the following theorem, which bounds the distance between q and the mean number of unmutated k-mers μ:

Theorem 2. *Fix $\alpha \in (0,1)$. Let X be a binomial random variable with probability of success p, n trials, and mean $\mu = nA^k$. Let q be the largest integer such that $\mathbb{P}(X \geq q) \geq \alpha$. Then there exists a positive constant C'_α depending only on α such that:*

$$\mu - C'_\alpha\sqrt{\mu} \leq q \leq \mu + C'_\alpha\sqrt{\mu}.$$

Proof. We can equivalently define q as the largest integer such that $\mathbb{P}(X < q) \leq 1 - \alpha$. By Theorem 1.2,

$$\mathbb{P}(X \leq (1-\delta)\mu) \leq \exp(-\mu\delta^2/2).$$

Setting $\exp(-\mu\delta^2/3) = 1 - \alpha$, we can compute that $\mathbb{P}(X \leq (1-\delta)\mu) \leq 1 - \alpha$ so long as

$$\delta \geq \sqrt{\frac{3\ln(1-\alpha)^{-1}}{\mu}}.$$

We conclude that

$$q \geq \left\lfloor \left(1 - \sqrt{\frac{3\ln(1-\alpha)^{-1}}{\mu}}\right)\mu \right\rfloor \geq \mu - C'_\alpha\sqrt{\mu}.$$

Similar logic for the upper tail gives $q \leq \mu + C'_\alpha\sqrt{\mu}$, possibly changing the constant C'_α. □

As a corollary, we can immediately infer that as n becomes large, q tends to be close to the mean μ:

Corollary 1. *$\mu/q \to 1$ as $n \to \infty$.*

We now prove a bound relating the alternative significance ANI a^α to the ANI threshold A:

[⋆] Alexei Novikov and Stephen White were partially supported NSF DMS-1813943. David Koslicki was supported by NSF award No. DMS-1664803 and the NIH grant 5R01GM146462-02

Theorem 3. *Fix $\alpha \in (0.5, 1)$ and let q be the largest integer such that $\mathbb{P}(X \geq q) \geq \alpha$ for $X \sim Binom(A^k, n)$. Let a^α be the number in $(0, A)$ such that for $Y \sim Binom((a^\alpha)^k, n)$, $\mathbb{P}(Y \geq q) = 1 - \alpha$. Then there exists a positive constant C'''_α depending only on α such that:*

$$\gamma_{n,k,A} A \leq a^\alpha \leq A \tag{1}$$

where

$$\gamma_{n,k,A} = \left(1 - \frac{C'''_\alpha}{\min\left\{nA^k, \sqrt{nA^k}\right\}}\right)^{1/k}.$$

In particular, as $n \to \infty$ with k fixed, $a^\alpha \to A$.

In words, this means that the statistical power of the test approaches 1 as the number of exclusive k-mers increases.

Proof. We have already concluded that $a^\alpha \leq A$, and so proving the result requires proving only the lower bound. To accomplish this, we set $\mu_\alpha = n(a^\alpha)^k$ and employ Theorem 1.1-3, which yields for $\delta \in (0, 1]$

$$\mathbb{P}\left(Y \geq (1+\delta)\mu_\alpha\right) \leq \exp\left(-\mu_\alpha \delta^2/3\right) \tag{2}$$

and when $\delta > 1$,

$$\mathbb{P}\left(Y \geq (1+\delta)\mu_\alpha\right) \leq \exp\left(-\mu_\alpha \delta/3\right). \tag{3}$$

We divide into the cases $\delta \leq 1$ and $\delta > 1$, which corresponds to the cases $(a^\alpha)^k \leq 2A^k$ and $(a^\alpha)^k > 2A^k$.

We begin with the case $(a^\alpha)^k \leq 2A^k$. By the change of variables $(1+\delta)\mu_\alpha \to q$, Theorem 1.2 gives:

$$\mathbb{P}\left(Y \geq q\right) \leq \exp\left(-\mu_\alpha (q/\mu_\alpha - 1)^2/3\right). \tag{4}$$

Next, we claim that for some positive C'''_α depending only on α,

$$\mu_\alpha \geq \mu - C'''_\alpha \sqrt{\mu}. \tag{5}$$

We consider two cases. First, suppose that $\mu_\alpha \geq q$. Then by theorem 2,

$$\mu_\alpha \geq \mu - C'_\alpha \sqrt{\mu}$$

so the claim holds with $C'''_\alpha = C'_\alpha$.

Next, we consider the case $\mu_\alpha < q$. We set the right-hand side of (4) to $1 - \alpha$, yielding

$$\mu_\alpha (q/\mu_\alpha - 1)^2 \leq 3 \ln(1-\alpha)^{-1}$$

which as an equation has two solutions for μ_α:

$$\mu_\alpha = q + \frac{3\ln(1-\alpha)^{-1}}{2} \pm \frac{\sqrt{12q\ln(1-\alpha)^{-1} + 9\ln(1-\alpha)^{-2}}}{2}.$$

Since $\mu_\alpha < q$ by assumption, we know that the lower bound for μ_α must be the smaller of these two solutions, as the other will be greater than q. Accordingly,

$$\mu_\alpha \geq q + \frac{3\ln(1-\alpha)^{-1} - \sqrt{12q\ln(1-\alpha)^{-1} + 9\ln(1-\alpha)^{-2}}}{2} \geq q - C''_\alpha \sqrt{q}$$

where C''_α is a constant depending only on α; thus claim (5) holds for all μ_α.

Substituting the result of theorem 2 into (5), we have

$$\mu_\alpha \geq (\mu - C'_\alpha \sqrt{\mu}) - C''_\alpha \sqrt{\mu + C'_\alpha \sqrt{\mu}} \geq \mu - C'''_\alpha \sqrt{\mu}$$

for new constant C'''_α depending only on α. Substituting $\mu_\alpha = n(a^\alpha)^k$ and $\mu = nA^k$, we have

$$(a^\alpha)^k \geq \left(1 - C'''_\alpha / \sqrt{nA^k}\right) A^k$$

and therefore

$$a^\alpha \geq A\left(1 - C'''_\alpha / \sqrt{nA^k}\right)^{1/k}. \tag{6}$$

as long as $(a^\alpha)^k \leq 2A^k$.

Following essentially the same steps in the case $(a^\alpha)^k > 2A^k$ and applying Theorem 4.1, we find that in this case

$$\mu_\alpha \geq q - 2\ln(1-\alpha)^{-1}$$

and thus that

$$a^\alpha \geq A\left(1 - \frac{C'''_\alpha}{nA^k}\right)^{1/k} \tag{7}$$

for possibly changed C'''_α. Taking the larger of the bounds from equations (6) and (7) completes the proof. □

Theorem 3 also suggests the somewhat surprising result that increasing k does not monotonically improve accuracy (in the sense of bringing a^α closer to A). The $\gamma_{n,k,A}$ term in line (1) has a unique global and local maximum for $k \geq 1$, suggesting there is an optimal k that maximizes the power of `YACHT` for a specific number of unique k-mers n. This phenomenon corresponds to the fact that for large k, nearly all of a genome's k-mers will be mutated at the ANI threshold A, so the hypothesis boundary q must be set very close to zero. When this happens, only small deviations are necessary for more highly mutated genomes to cross the inclusion threshold. This behavior is reflected in the following section in Figure S3.

The critical point could be estimated theoretically using the estimates of 3, but as these are only approximations it will be more practical to simply test a range of k-mer values with the exact binomial CDF. It should also be noted that this analysis assumes n remains unchanged as k increases, when in practice the number of k-mers unique to each organism can be expected to increase with k as overlaps become less probable, which would partially mitigate the downside of increased k in real-world applications.

S2 Synthetic Data-based Experiments

In this section, we describe a series of experiments on synthetic data to aid in understanding the impact on performance of various model parameters.

S2.1 Synthetic Data

In this section, we show results of the `YACHT` method using simulated data. The simulation parameters are detailed in Table 1. When not specified as part of the experiment, the default parameters are used. We note that our synthetic data always has in-sample coverage of 1; see the main text for experiments verifying performance when true coverage is less than one. All simulations were run for 100 iterations for each set of parameters.

Parameter Name	Symbol	Description	Default
`--ksize`	k	k-mer size	31
`--num_kmers`	n	Number of k-mers per genome	1000
`--num_genomes`	N	Number of genomes in reference	1000
`--s_known`	s_k	Number of true known organisms in sample	100
`--s_unknown`	s_u	Number of true unknown organisms in sample	100
`--ani_thresh`	A	Mutation rate cutoff for known/unknown	0.95
`--relation_thresh`	r	Maximum ANI between reference genomes	0.95
`--significance`	α	Significance level for testing	0.99
`--min_coverage`	C	Minimum coverage for significance level	1
`--ani_range`	[m,M]	Range of possible ANI's	[0.9,1]

Table 1: Synthetic data simulation parameters.

We note that we have set the default minimum ANI to 0.9, which is much lower than the largest mutation rates seen in practice. As will be shown, YACHT only experiences false positives relatively close to the ANI cutoff A, so a low upper bound was chosen to ensure a nontrivial number of false positives in the resulting simulations. The choice of 0.9 exactly allows for symmetric distribution of mutation rates around the ANI threshold $A = 0.95$. As a result of this choice, the observed false positive rates are inflated relative to what might be expected in practice, where the lowest true ANI's will be closer to 0.7. To aid in interpretation of our numerical results, roughly speaking, in this setting a 0.2 false positive rate corresponds to accepting false positives with true ANI as low as 0.94, while a 0.5 false positive rate would correspond to accepting false positives with true ANI as low as 0.925.

Simulated Reference Model We employ the following model for the reference genomes $\mathcal{G}$. The total number of k-mers across all genomes is set at $K = nN(1 - r^k)$ (rounded to the nearest integer). The first genome G_1 consists of n randomly selected k-mers, while subsequent genomes G_i consist of $\lfloor nr^k \rfloor$ k-mers chosen uniformly from G_{i-1} and $n - \lfloor nr^k \rfloor$ chosen uniformly from its complement. This model guarantees each genome in $\mathcal{G}$ is highly related to at least one other genome in the reference with an ANI of r.

All simulations are run under this model except for runtime/memory simulations, which use a similar but deterministic model for the reference which can be generated more quickly. This does not affect the recorded recovery runtimes, which do not take into account the time spent setting up the synthetic data.

Simulated Sample Model The sample $\mathcal{S}$ is constructed according to the random sample model in the main text Definition 2 with randomized mutation rates $\mathbf{r}$ set as follows. Out of the N genomes, a uniformly random set of s_k genomes is labeled known and each chosen genome is assigned an independent and uniformly random ANI in the interval $[A, M]$. Likewise, s_u genomes are randomly chosen to be unknown and each assigned an independent and uniformly random ANI in $[m, A]$. The remainder of the genomes in $\mathcal{G}$ are omitted from the sample (or, equivalently, assigned an ANI of 0).

S2.2 Significance level reflects true false negative rate

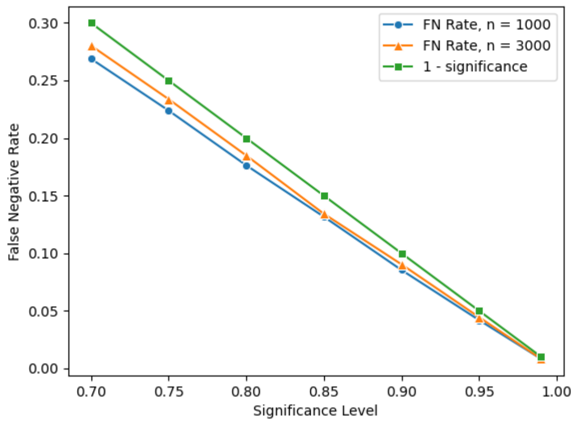

Fig. S1: **False negative rate vs. significance level.** The vertical axis gives the number of false negatives divided by the total number of known organisms over 100 iterations. The horizontal axis gives the value of the user-specified significance level α.

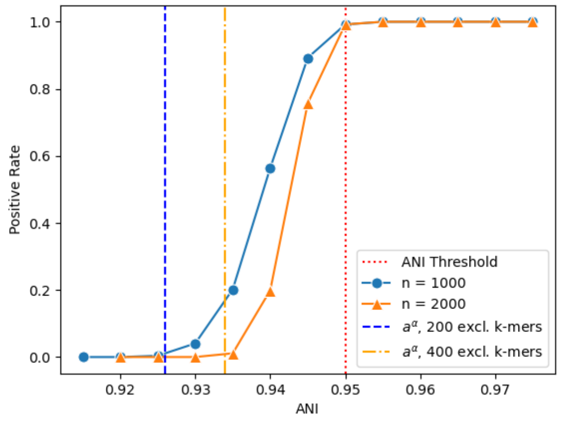

Fig. S2: **Positive rate vs. ANI.** The vertical/y-axis gives the number of organisms marked present divided by the total number of organisms in sample over 100 iterations. The horizontal/x-axis gives the true ANI rate of each genome in the sample to a genome in the reference. The left-most and middle vertical dashed lines give the alternative significance ANI using 200 and 400 exclusive k-mers respectively; the right-most vertical dashed line gives the ANI threshold.

Figure S1 shows the true false negative rate out of 200 known organisms when mutation rate is fixed at exactly 0.05. We see that false negative rate is always slightly less than 1 minus the specified significance level. As the binomial distribution is discrete, the hypothesis boundary cannot be chosen exactly to result in a false negative rate of $1 - \alpha$, and so is always an underestimate. Comparing the results for $n = 1000$ and $n = 3000$, we see that increasing the number of k-mers per genome reduces this difference.

S2.3 Prediction Rate vs. ANI

In Figure S2, we show the rate of positive results (organisms marked present in the sample) versus the underlying mutation rate. For this experiment, 200 organisms were in the sample with true mutation rates *exactly* equal to a specified ANI which was varied from 0.92 to 0.97. We see that for ANI substantially below the ANI threshold, the positive rate is 0, before quickly increasing to 1 above the threshold. We also observe that the transition from 0 to

1 occurs more quickly for $n = 2000$ than $n = 1000$, reflecting the increase in statistical power that accompanies a greater number of k-mers per organism. The blue and orange vertical lines show the alternative significance ANI (a^α) for 200 and 400 exclusive k-mers, respectively, showing that a greater number of k-mers corresponds to a^α closer to the ANI threshold.

S2.4 Performance in k reflects theoretical predictions

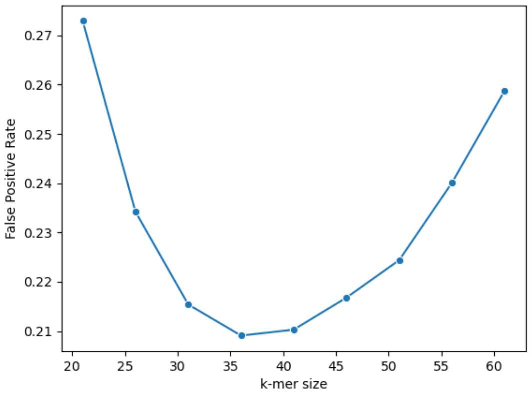

(a) **False positive rate vs. k-mer size** The vertical/y-axis shows fraction of false positives out of sample organisms when setting ANI threshold to 0.95. The horizontal/x-axis shows k-mer size k.

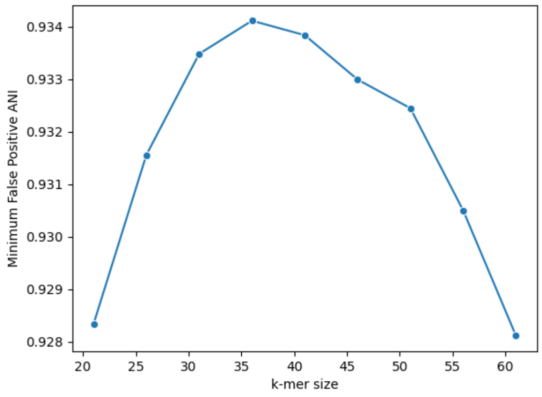

(b) **Minimum false positive ANI vs. k-mer size.** The vertical/y-axis shows the minimum ANI of the false positives, averaged over 100 trials. The horizontal/x-axis shows k-mer size k.

Fig. S3: **False positive performance vs. k-mer size.** Figure S3a (left) shows the effect of k-mer size on false positive rate, while Figure S3b (right) shows its effect on minimum false positive ANI.

Figure S3 shows performance as k-mer size is increased. As an estimate for a^α for each simulated reference and sample, the minimum ANI among false positive results was tracked (with the ANI threshold A substituted in this average in the case of no false positives). Figure S3b shows the average of these minimum false positive ANI's across 100 simulations for varying k-mer sizes k. As predicted by the $\gamma_{n,k,A}$ term from Theorem 3, the minimum false positive ANI initially rises to a local maximum in k before decreasing once k gets too large. A similar pattern can be observed in the false positive rate in figure S3a.

S2.5 Runtime and space usage

In Figures S4-S5, we show how runtime and space usage vary with the two main parameters which affect it, number of k-mers per reference genome n and number of reference genomes N. Growth in runtime is at-most linear in both n and N, though the growth of runtime in n is somewhat inconsistent over small changes. By contrast, memory usage grows nearly exactly linearly in both variables.

S2.6 Performance with Varying Parameters

In this section, we show how false positive and false negative rate are affected by changing various simulation parameters.

N: Number of genomes in reference (figure omitted). In experiments with synthetic data, changing the number of genomes in the reference had essentially no effect on false positive or false negative rate. This is expected due to the use of exclusive k-mers implying each genome tested is independent from all others.

c: Minimum coverage for significance level. In Figure S7, we show how false positive rate changes with minimum coverage parameter C. As expected—given that true coverage remains unchanged at 1—a lower minimum coverage results in higher false positive rate.

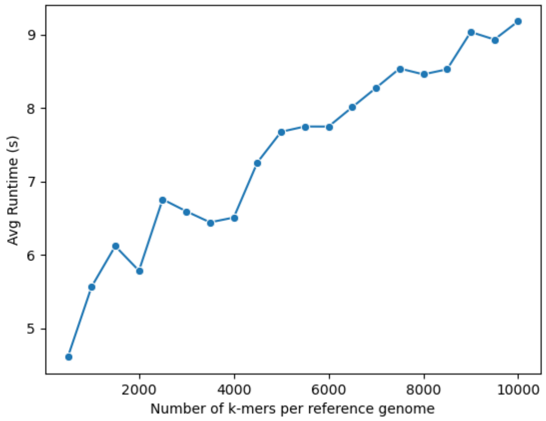

(a) **Recovery runtime vs. number of k-mers per reference genome.** The horizontal/y-axis shows average runtime of the recovery process in seconds. The horizontal/x-axis shows the number of k-mers per reference genome.

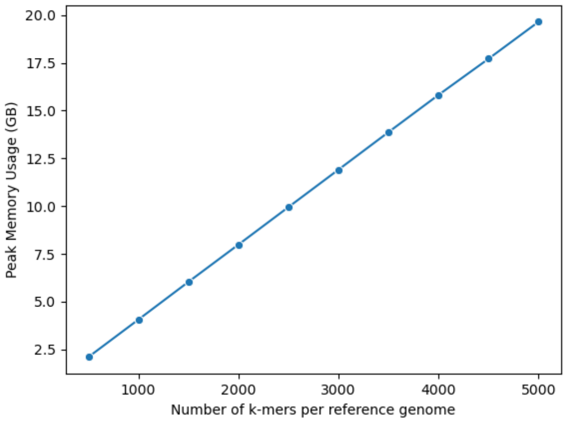

(b) **Maximum memory usage vs. number of k-mers per reference genome.** The horizontal/y-axis shows maximum memory usage in GB. The horizontal/x-axis shows the number of k-mers per reference genome.

Fig. S4: **Runtime and memory usage vs. number of k-mers per reference genome.** On the left, figure S4a shows the effect of number of k-mers per reference genome on recovery runtime while figure S4b on the right shows its effect on maximum memory usage.

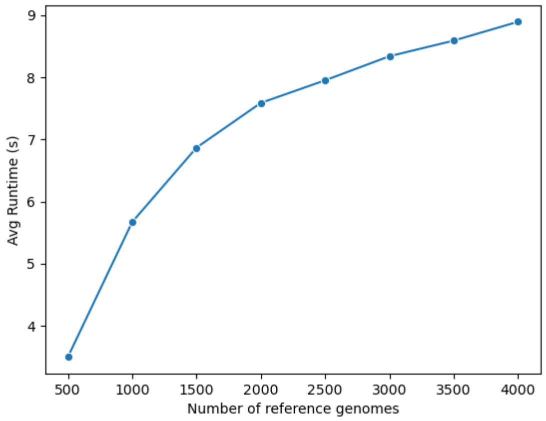

(a) **Recovery runtime vs. number of reference genomes.** The horizontal/y-axis shows average runtime of the recovery process in seconds. The horizontal/x-axis shows the number of genomes in the reference dictionary.

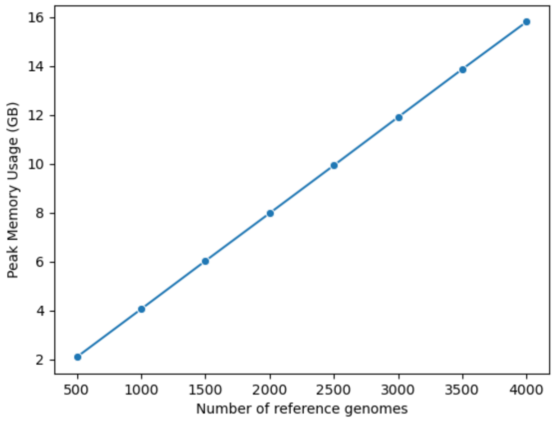

(b) **Recovery memory usage vs. number of reference genomes.** The horizontal/y-axis shows maximum memory usage in GB. The horizontal/x-axis shows the number of genomes in the reference dictionary.

Fig. S5: **Runtime and memory usage vs. number of reference genomes.** On the left, figure S5a shows the effect of number of reference genomes on recovery runtime while figure S5b on the right shows its effect on maximum memory usage.

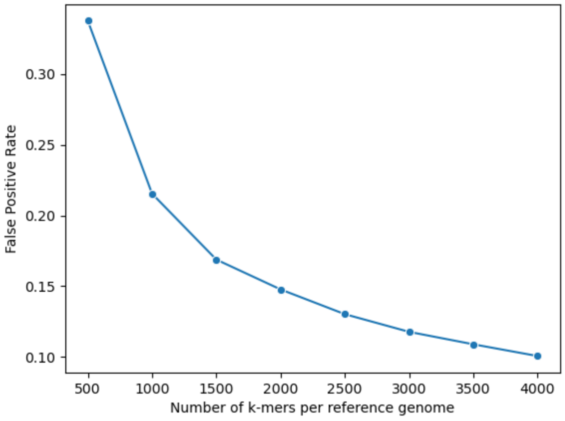

Fig. S6: **False positive rate vs. number of k-mers per reference genome.** The vertical/y-axis shows fraction of false positives out of sample organisms when setting the ANI threshold to 0.95. The horizontal/x-axis shows number of k-mers per genome in the reference dictionary.

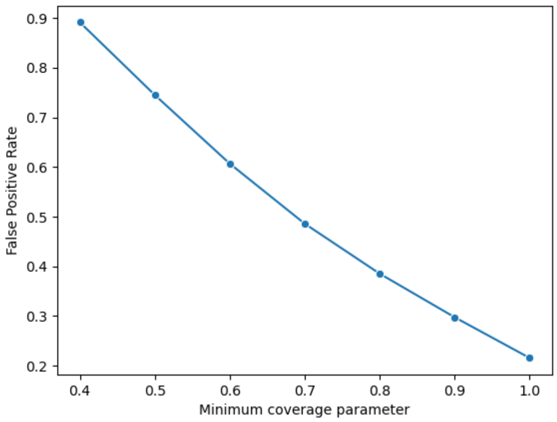

Fig. S7: **False positive rate vs. `--min_coverage` parameter** The vertical/y-axis shows fraction of false positives out of sample organisms when setting the ANI threshold to 0.95. The horizontal/x-axis shows user-specified `--min_coverage` parameter.

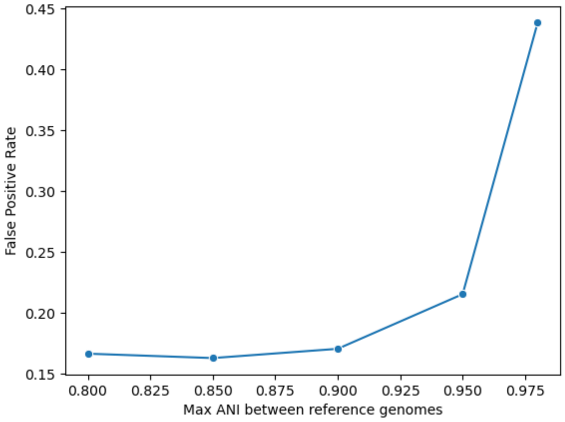

Fig. S8: **False positive rate vs. max ANI between reference genomes.** The vertical/y-axis shows fraction of false positives out of sample organisms when setting the ANI threshold to 0.95. The horizontal/x-axis shows the maximum ANI between reference genomes.

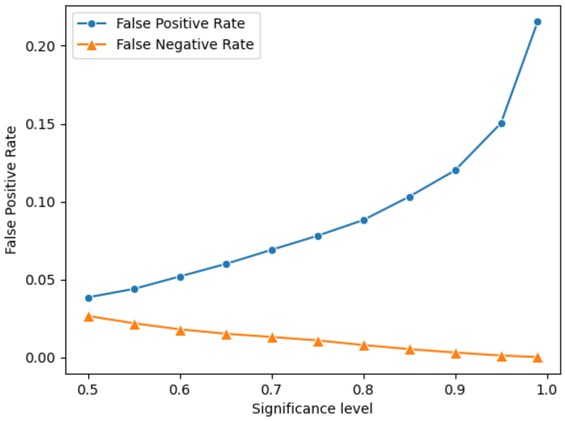

Fig. S9: **False positive/negative rates vs. significance level.** The vertical/y-axis shows fraction of false positives and false negatives out of sample organisms with ANI under (respectively, above) threshold 0.95. The horizontal/x-axis shows the user-specified significance level α.

r: Maximum ANI between reference genomes. Figure S8 shows that as the ANI between reference genomes increases, so does false positive rate. An especially dramatic spike occurs when ANI exceeds the ANI threshold A, as this drastically reduces the number of k-mers unique to each genome in the reference.

α: Significance level. Figure S9 demonstrates the tradeoff between false positive and false negative rate that is controlled by α. When $\alpha = 0.5$, false positive and false negative rates are nearly equal; as α grows to 0.99, false negative rate declines nearly to zero while false positive rate becomes high.

S3 Data Pre-processing Details for Different Experiments

S3.1 Spike-in Experiments

Reference data We collected 1,044 bacterial genomes randomly selected from NCBI RefSeq [2] to build the reference database. These genomes were processed using sourmash's `index` with a k-mer size of 31 and a scale factor of $1,000$ (i.e., $1/1,000$ of all sketches of a genome) and the YACHT preprocessing Algorithm 4 with the ANI threshold $A = 0.95$.

Real metagenome We selected a random, publicly available human gut metagenome from the ENA [3] which we call the **real metagenome**. Specifically, from the project PRJEB9576 we selected the Illumina HiSeq 2000 sample with accession number SAMEA3449194 and took the forward reads only, for a total of 27M reads. We utilized sourmash's `gather` metagenome profiler to predict which genomes from our reference database are present in this metagenome. Importantly, we set the parameter `--coverage-bp` to zero (i.e. maximum sensitivity) in order to detect the most reference genomes present in this metagenome. A total of 268 reference database genomes were detected as being "present" in this metagenome and we refer to the remaining 776 reference database genomes as **absent reference genomes** as they are, with high confidence, not present in the metagenome.

Known unknowns In order to test the `--ani_thresh` parameter, we need to collect genomes that are not contained in the reference data, but are at a known ANI to genomes that are in the reference. To that end, we downloaded the GTDB [4,5] R07-RS207 genomic representatives [1] database which contains 65,703 genomes. After forming FracMinHash sketches, we utilized the approach of [6] via sourmash to compute the ANI of each of these 65K genomes to the absent reference genomes described in the previous paragraph, retaining those with an ANI above 0.7 to any of the absent reference genomes. We then selected from these those genomes that were absent from the real metagenome in the exact same way as in the previous paragraph. This resulted in a total of 28,595 **known unknowns**: genomes that are neither in the sample nor in the reference, but are at a known ANI to genomes in the reference.

S3.2 CAMI II based Experiments

Reference data We built two different reference databases respectively for the taxonomic profiling and the pathogen detection in the CAMI II challenge [7]. For taxonomic profiling challenge, we downloaded the NCBI RefSeq database provided by CAMI II [2] which contains 141,677 genomes. After filtering out those without taxids and those whose taxids are not "species" or "strain", we merged the remaining genomes with the genomes used in the CAMI II taxonomic profiling challenge (e.g., marine, rhizosphere, and strain madness datasets)[3]. This resulted in a total of 89,898 genomes for building reference database. For pathogen detection challenge, we respectively collected the bacterial and viral reference genomes from the Bacterial and Viral Bioinformatics Resource Center (BV-BRC) [8], and fungal genomes from NCBI RefSeq and Genbank. Again, we removed genomes absent of taxids or not labeled as "species" or "strain", and finally had 30,091 reference genomes (8,970 bacteria, 16,077 viruses, 5,044 fungi) for the pathogen detection reference database. All reference genomes were converted to hashes via sourmash's `index` with a k-mer size of 31 and a scale factor of 100 (i.e., 1/100 of all sketches of a genome) and pre-processed using the Algorithm 4.

Metagenome samples We utilized the CAMI II metagenome samples: 10 marine, 1 pathogen detection, 21 rhizosphere, and 100 strain madness datasets. These samples were downloaded via CAMI II portal (see footnote 3). They were indexed via sourmash's `index` with a k-mer size of 31 and a scale factor of 100 .

[1] via https://data.gtdb.ecogenomic.org/releases/release207/207.0/genomic_files_reps/gtdb_genomes_reps_r207.tar.gz

[2] via https://openstack.cebitec.uni-bielefeld.de:8080/swift/v1/CAMI_2_DATABASES/RefSeq_genomic_20190108.tar

[3] data downloaded via https://data.cami-challenge.org/participate

S3.3 Real-world Data-based Experiment

Reference data We utilized the same reference database as we used in the pathogen detection task in the CAMI II challenge (see description above S3.2).

Metagenome samples We selected the metagenome samples from a publication [9] where they provide a mock bacterial community stool sample with 10 known bacterial species and eight individual stool samples spiked with proportions of the mock community with those 10 known bacteria. These samples were downloaded from the NCBI Sequence Read Archive (SRA) with accession numbers: ERR1971004, ERR1971008, ERR1971003, ERR1971007, ERR1971009, ERR1971006, ERR1971005, ERR1971010, ERR1971013. The sample ERR1971006 was excluded due to file corruption, and the sample ERR1971013 is the mock community sample. All these samples were processed with the same fashion as we used in the previous two experiments (described above) via sourmash's `index` and the Algorithm 4.

S4 Comparison with sourmash

The tool sourmash [10] possesses a sub-routine called `prefetch` which performs organism presence/absence detection via FracMinHash calculated containment indices. sourmash's subsequent sub-routine `gather` then uses a minimum set cover approach to reduce the number of false positives. In this section, we compare the performance of this presence/absence approach versus `YACHT`.

Due to needing a ground truth in order to assess performance, we simulated data from the real genomes. For the sample metagenomes, we randomly selected 200 genomes from the reference database used in the spike-in experiments described in and used `BBMap` [11] to generate a metagenome from them consisting of 10 million noisy reads. To mimic the situation where the metagenome contains novel genomes not present in the reference database, we then removed half (100) of these genomes from the reference database before running sourmash `prefetch` and `gather` (with a `--threshold-bp` value of 100 and all other parameters at their defaults) and `YACHT` (with a `--min_coverage` value of 1 and all other parameters at their defaults). This procedure was then repeated a total of 30 times each for two different distributions of genome relative abundance: uniform and exponential distributions. The resulting false positive and negative rates are depicted in Figure S10. As can be seen from this figure, in comparison to sourmash, `YACHT` experiences significantly fewer false positives and equivalent false negatives in the uniform distribution case, or slightly increased false negatives in the exponential case. This later case of increased false negatives is due to the exponential distribution causing low abundance genomes to have a coverage in the sample well below the `--min_coverage` value of 1, similar to what was seen in Figure 2 in main text.

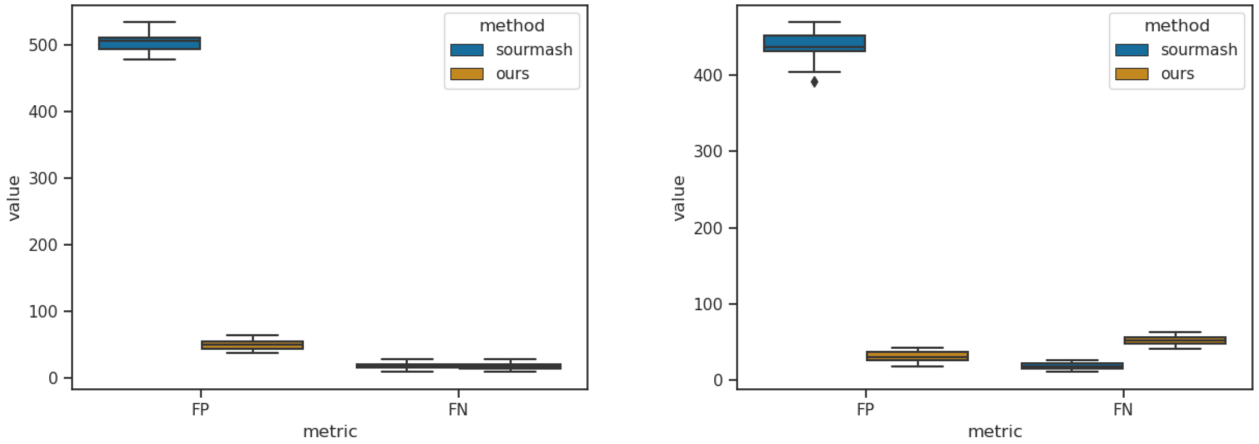

(a) **Uniform distribution of relative abundance.** (b) **Exponential distribution of relative abundance.**

Fig. S10: **False positive and negative rates of `YACHT` vs sourmash.** Performance is measured on 30 replicates of simulated metagenomes consisting of 10 million noisy reads generated from 200 randomly selected genomes, half of which were removed from the reference database prior to running sourmash and `YACHT`. The relative abundance of these 200 genomes was pulled from either (a) a uniform or (b) exponential distribution.

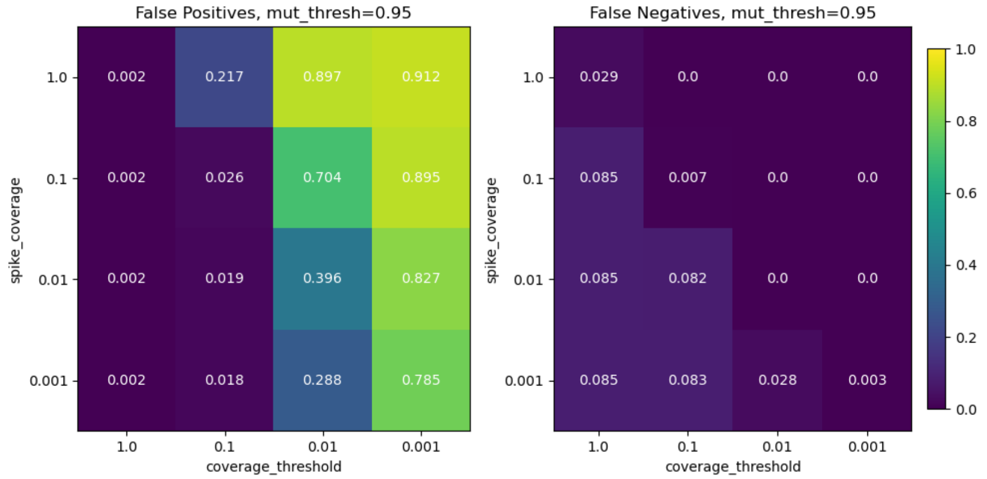

(a) **Parameter --ani_thresh set to 0.95**

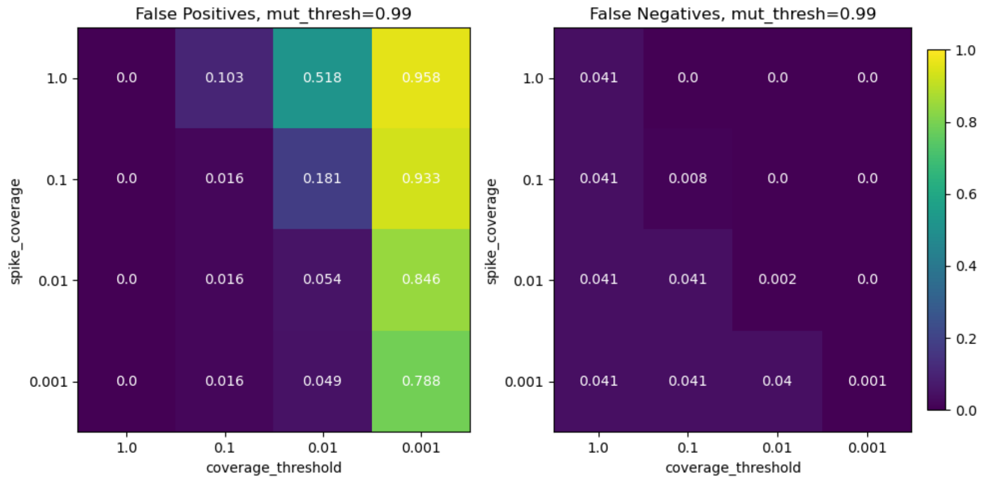

(b) **Parameter --ani_thresh set to 0.99**

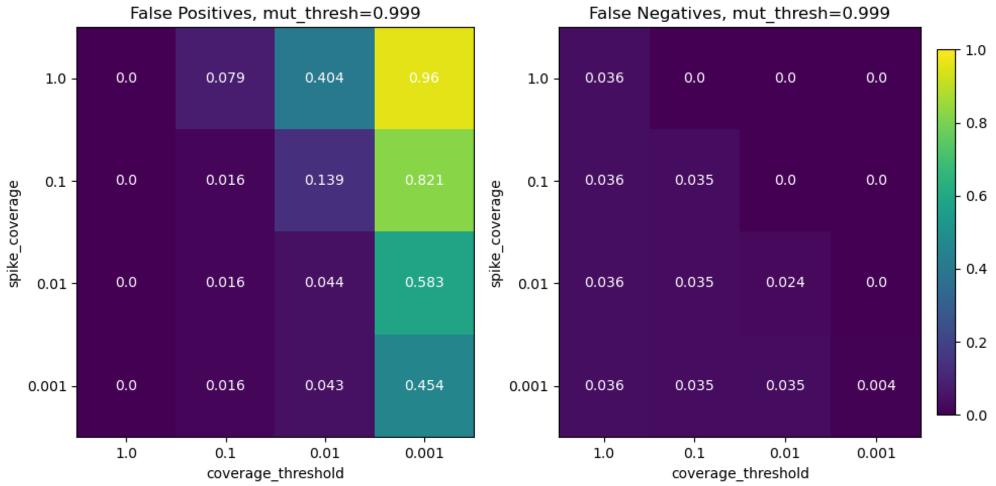

(c) **Parameter --ani_thresh set to 0.999**

Fig. S11: **False positive and negative rates for spike in experiments.** The heatmaps on the left show the False positive rate and the ones on the right show the false negative rate when the `--ani_thresh` parameter was set to (a) 0.95, (b) 0.99 and (c) 0.999. In each heat map, the horizontal axis varies the coverage threshold parameter C and the vertical axis varies the coverage c of the spiked in genome in the metagenome.

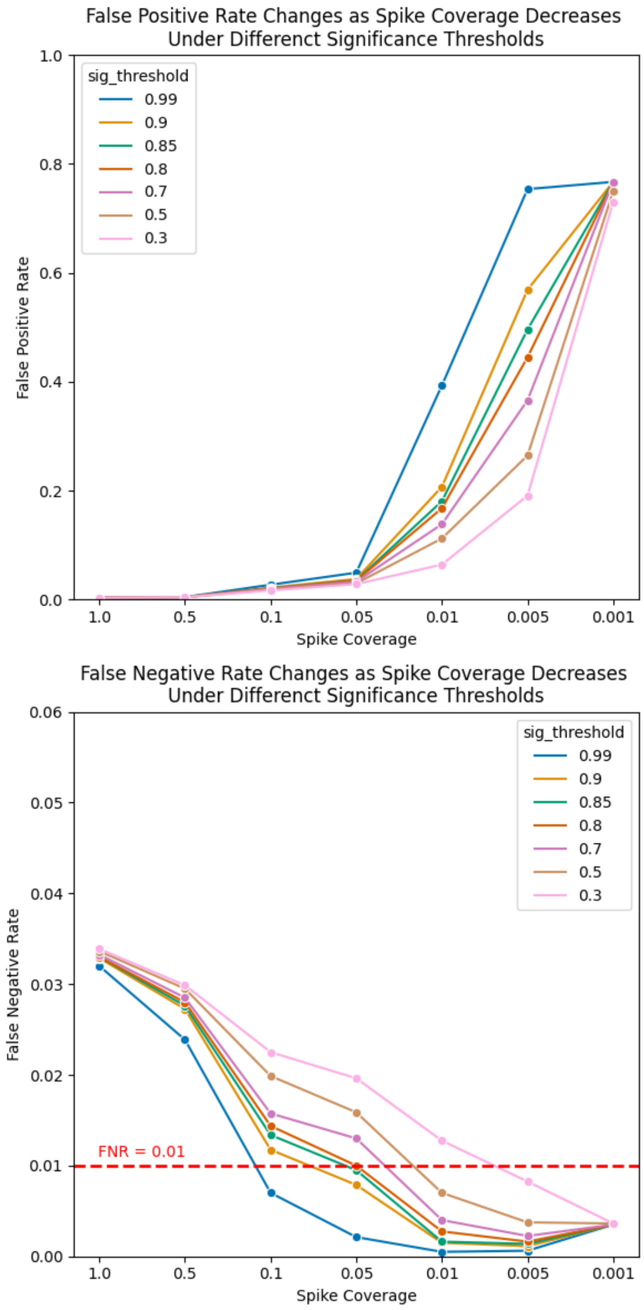

Fig. S12: Line plots of false positive rate and false negative rate versus spike coverage under different significance thresholds when setting the ANI threshold to 0.95. In each plot, the horizontal axis varies the coverage c of the spiked in genome in the metagenome (fixing the coverage threshold C to c) while the vertical axis respectively represents false positive rate and false negative rate. We used different color lines to present different significance threshold settings. In the false positive rate plot, we highlighted the line with FNR $= 0.01$ with red dash line. Note the different y-axes scales in the two figures.

S5 Additional Results

Figure S11, as a supplementary figure for Figure 4 in the main text, shows the result of these experiments when c and C both vary over 4 orders of magnitude, from 1.0 to 0.001.

Figure S12 indicates the changes of false positive rate (FPR) and False negative rate (FNR) when c and C both vary over 4 orders of magnitude, from 1.0 to 0.001, under different significance thresholds (α).

References

1. M. Mitzenmacher and E. Upfal, *Probability and Computing: Randomized Algorithms and Probabilistic Analysis*. USA: Cambridge University Press, 2005.
2. N. A. O'Leary, M. W. Wright, J. R. Brister, S. Ciufo, D. Haddad, R. McVeigh, B. Rajput, B. Robbertse, B. Smith-White, D. Ako-Adjei *et al.*, "Reference sequence (refseq) database at ncbi: current status, taxonomic expansion, and functional annotation," *Nucleic acids research*, vol. 44, no. D1, pp. D733–D745, 2016.
3. R. Leinonen, R. Akhtar, E. Birney, L. Bower, A. Cerdeno-Tárraga, Y. Cheng, I. Cleland, N. Faruque, N. Goodgame, R. Gibson *et al.*, "The european nucleotide archive," *Nucleic acids research*, vol. 39, no. suppl_1, pp. D28–D31, 2010.
4. P.-A. Chaumeil, A. J. Mussig, P. Hugenholtz, and D. H. Parks, "Gtdb-tk: a toolkit to classify genomes with the genome taxonomy database," 2020.
5. D. H. Parks, M. Chuvochina, C. Rinke, A. J. Mussig, P.-A. Chaumeil, and P. Hugenholtz, "Gtdb: an ongoing census of bacterial and archaeal diversity through a phylogenetically consistent, rank normalized and complete genome-based taxonomy," *Nucleic acids research*, vol. 50, no. D1, pp. D785–D794, 2022.
6. M. R. Hera, N. T. Pierce-Ward, and D. Koslicki, "Debiasing fracminhash and deriving confidence intervals for mutation rates across a wide range of evolutionary distances," *bioRxiv*, 2022.
7. F. Meyer, A. Fritz, Z.-L. Deng, D. Koslicki, T. R. Lesker, A. Gurevich, G. Robertson, M. Alser, D. Antipov, F. Beghini *et al.*, "Critical assessment of metagenome interpretation: the second round of challenges," *Nature methods*, vol. 19, no. 4, pp. 429–440, 2022.
8. R. D. Olson, R. Assaf, T. Brettin, N. Conrad, C. Cucinell, J. J. Davis, D. M. Dempsey, A. Dickerman, E. M. Dietrich, R. W. Kenyon *et al.*, "Introducing the bacterial and viral bioinformatics resource center (bv-brc): a resource combining patric, ird and vipr," *Nucleic acids research*, vol. 51, no. D1, pp. D678–D689, 2023.
9. P. I. Costea, G. Zeller, S. Sunagawa, E. Pelletier, A. Alberti, F. Levenez, M. Tramontano, M. Driessen, R. Hercog, F.-E. Jung *et al.*, "Towards standards for human fecal sample processing in metagenomic studies," *Nature biotechnology*, vol. 35, no. 11, pp. 1069–1076, 2017.
10. L. C. Irber, P. T. Brooks, T. E. Reiter, N. T. Pierce-Ward, M. R. Hera, D. Koslicki, and C. T. Brown, "Lightweight compositional analysis of metagenomes with fracminhash and minimum metagenome covers," *bioRxiv*, 2022.
11. B. Bushnell, "Bbmap: a fast, accurate, splice-aware aligner," Lawrence Berkeley National Lab.(LBNL), Berkeley, CA (United States), Tech. Rep., 2014.
